# Rapid and stable mobilization of CD8^+^ T cells by SARS-CoV-2 mRNA vaccine

**DOI:** 10.1038/s41586-021-03841-4

**Published:** 2021-07-28

**Authors:** Valerie Oberhardt, Hendrik Luxenburger, Janine Kemming, Isabel Schulien, Kevin Ciminski, Sebastian Giese, Benedikt Csernalabics, Julia Lang-Meli, Iga Janowska, Julian Staniek, Katharina Wild, Kristi Basho, Mircea Stefan Marinescu, Jonas Fuchs, Fernando Topfstedt, Ales Janda, Oezlem Sogukpinar, Hanna Hilger, Katarina Stete, Florian Emmerich, Bertram Bengsch, Cornelius F. Waller, Siegbert Rieg, Sagar, Tobias Boettler, Katharina Zoldan, Georg Kochs, Martin Schwemmle, Marta Rizzi, Robert Thimme, Christoph Neumann-Haefelin, Maike Hofmann

**Affiliations:** 1 Department of Medicine II (Gastroenterology, Hepatology, Endocrinology and Infectious Diseases), Freiburg University Medical Center, Faculty of Medicine, University of Freiburg, Freiburg, Germany; 2 Faculty of Biology, University of Freiburg, Freiburg, Germany; 3 IMM-PACT, Faculty of Medicine, University of Freiburg, Freiburg, Germany; 4 Institute of Virology, Freiburg University Medical Center, Faculty of Medicine, University of Freiburg, Freiburg, Germany; 5 Department of Rheumatology and Clinical Immunology, Freiburg University Medical Center, Faculty of Medicine, University of Freiburg, Freiburg, Germany; 6 Faculty of Chemistry and Pharmacy, University of Freiburg, Freiburg, Germany; 7 Department of Pediatrics and Adolescent Medicine, Ulm University Medical Center, Ulm, Germany; 8 Institute for Transfusion Medicine and Gene Therapy, Freiburg University Medical Center, Faculty of Medicine, University of Freiburg, Freiburg, Germany; 9 Signalling Research Centres BIOSS and CIBSS, University of Freiburg, Freiburg, Germany; 10 Department of Haematology, Oncology & Stem Cell Transplantation, Freiburg University Medical Center, Faculty of Medicine, University of Freiburg, Freiburg, Germany; 11 Berta-Ottenstein Programme, Faculty of Medicine, University of Freiburg, Freiburg, Germany

**Keywords:** Immunological memory, Lymphocyte differentiation, Viral infection, RNA vaccines, SARS-CoV-2

## Abstract

SARS-CoV-2 spike mRNA vaccines^1–3^ mediate protection from severe disease as early as ten days after prime vaccination^3^, when neutralizing antibodies are hardly detectable^4–6^. Vaccine-induced CD8^+^ T cells may therefore be the main mediators of protection at this early stage^7,8^. The details of their induction, comparison to natural infection, and association with other arms of vaccine-induced immunity remain, however, incompletely understood. Here we show on a single-epitope level that a stable and fully functional CD8^+^ T cell response is vigorously mobilized one week after prime vaccination with bnt162b2, when circulating CD4^+^ T cells and neutralizing antibodies are still weakly detectable. Boost vaccination induced a robust expansion that generated highly differentiated effector CD8^+^ T cells; however, neither the functional capacity nor the memory precursor T cell pool was affected. Compared with natural infection, vaccine-induced early memory T cells exhibited similar functional capacities but a different subset distribution. Our results indicate that CD8^+^ T cells are important effector cells, are expanded in the early protection window after prime vaccination, precede maturation of other effector arms of vaccine-induced immunity and are stably maintained after boost vaccination.

## Abstract (web summary)

Longitudinal analyses of SARS-CoV-2 mRNA vaccine-elicited epitope-specific CD8^+^ T cell responses shows that CD8^+^ T cells are rapidly induced after prime vaccination and stably maintained after boost vaccination.

## Main

The current SARS-CoV-2 vaccination campaign provides the unique opportunity to gain important insights into human CD8^+^ T cell biology in the context of prime or boost mRNA vaccination. Initial data revealed that all arms of adaptive immunity such as neutralizing antibodies, virus-specific CD4^+^ T cells with T helper 1 (T_H_1) polarization and IFNγ-producing CD8^+^ T cells emerge after prime or boost vaccination^4,5,9^. The onset of mRNA vaccine-mediated protection has been observed as early as 10–12 days after the first dose^3^. During this early phase, T cells and spike-specific antibodies are detectable^7,8^, whereas neutralizing antibodies first appear after boost^4–6,10,11^. These observations point towards a key role of vaccine-induced T cells in early protection after prime vaccination. Previous studies focused on the analysis of the overall vaccine-elicited spike-reactive T cell response^4,5,7,8,12^; however, by this approach, the strength, dynamics and functional capacity are underestimated or even blurred in contrast to analyses performed at the single epitope level^5^. Here, we conducted continuous longitudinal analyses starting at baseline of prime vaccination until 3–4 months after boost on a single epitope level, to track the trajectories of bnt162b2 vaccine-elicited spike-specific CD8^+^ T cell responses in comparison to spike-specific CD4^+^ T cells, B cells, antibodies and their neutralizing activity.

## Vaccine-elicited CD8^+^ T cells

**Fig. 1:**
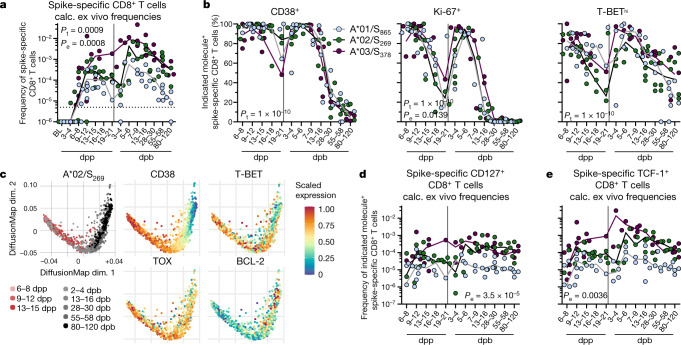
Vaccine-elicited epitope-specific CD8^+^ T cells. **a**, Calculated ex vivo frequency indicated at baseline (BL), dpp and dpb for spike-specific CD8^+^ T cells. Detection limit: 5 × 10^−6^. **b**, Percentage of CD38, Ki-67 and T-BET^hi^ expressing spike-specific non-naive CD8^+^ T cells. **c**, Diffusion map showing flow cytometry data for A*02/S_269_-specific CD8^+^ T cells in relation to dpp (shades of red) and dpb (shades of grey) in one individual. Expression levels of CD38, T-BET, TOX and BCL-2 are plotted on the diffusion map (blue denotes low expression; red denotes high expression). **d**, **e**, Calculated ex vivo frequencies of non-naive spike-specific CD8^+^ T cells expressing CD127 or TCF-1 for spike-specific CD8^+^ T cells. Line indicates median. *P* values determined by two-way ANOVA with main effects only comparing the effect of the different epitopes (*P*_e_) and of time course (*P*_t:_). Source data

**Extended Data Fig. 1:**
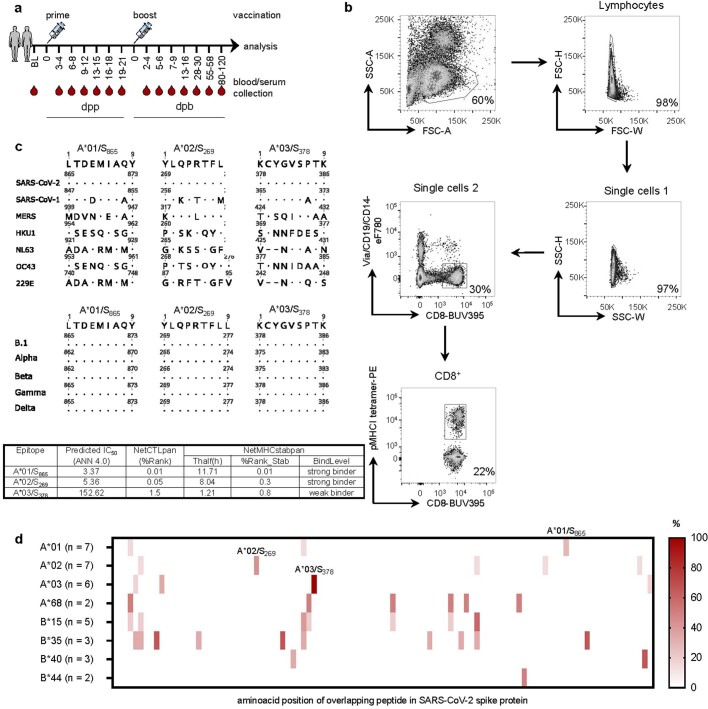
Spike-specific CD8+ T cell epitopes following vaccination. **(a)** Timeline showing blood and serum collection before and after prime and boost vaccination. **(b)** Gating strategy of flow cytometry data. A*01/S_865_-, A*02/S_269_- and A*03/S_378_-specific CD8+ T cells were identified via pMHCI tetramer-based analysis. **(c)** Comparison of epitope sequences with amino acid sequences of SARS-CoV-1/2, MERS and common cold coronaviruses amino acid sequences (upper panel) and with circulating SARS-CoV-2 variants of concern (VOC) (middle panel), respectively for A*01/S_865_, A*02/S_269_ and A*03/S_378_-specific CD8+ T cell epitopes. (Lower panel) A*01/S_865_, A*02/S_269_ and A*03/S_378_ peptide characteristics, comparing different prediction methods for the estimation of MHC I binding affinity, half-life and processing. **(d)** Heatmap showing the percentage of patients with a CD8+ T cell response to spike overlapping peptides/fine-mapped minimal optimal epitope in relation to the total number of patients tested with the respective HLA type. *n* = 16 OLP: overlapping peptide. Parts of the figure were drawn by using pictures from Servier Medical Art (http://smart.servier.com/) and licensed under a Creative Common Attribution 3.0 Generic License (a). Source data

**Extended Data Fig. 2:**
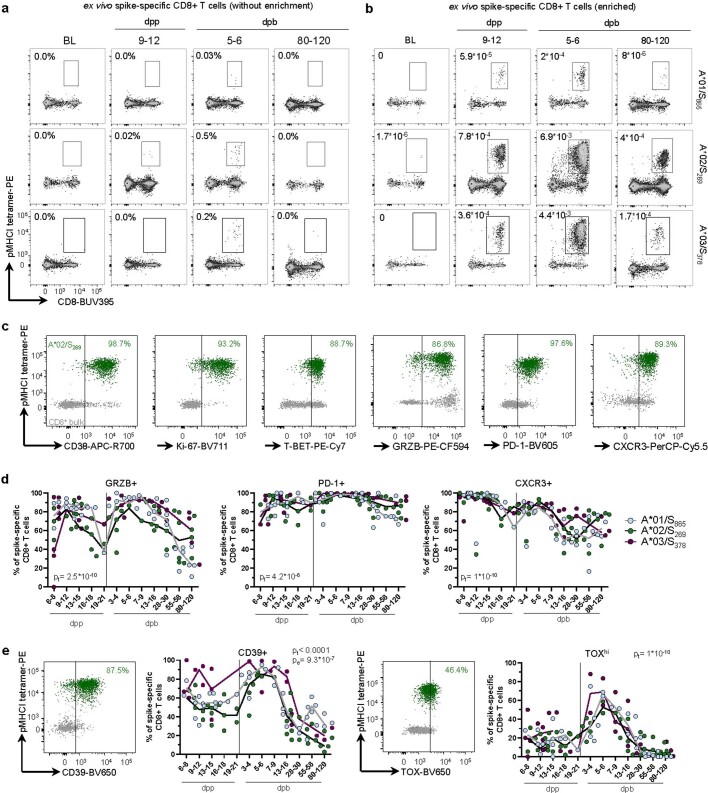
Spike-specific CD8+ T cells before and after enrichment. **(a, b)** Dot plots showing A*01/S_865_-, A*02/S_269_- and A*03/S_378_-specific CD8+ T cells ex vivo without pMHCI tetramer-based enrichment **(a)** and after pMHCI tetramer-based enrichment **(b)** at BL, before and after boost vaccination. **(c)** Exemplary dot plots (5-6 dpb) depicting the expression levels of CD38, Ki-67, T-BET, GRZB, PD-1 and CXCR3 in A*02/S_269_- (green) specific and bulk (grey) CD8+ T cells. **(d)** % of GRZB, PD-1 and CXCR3 expressing A*01/S_865_-, A*02/S_269_- and A*03/S_378_-specific non-naïve CD8+ T cells. **(e)** Exemplary dot plot (5-6 dpb) depicting the expression levels of CD39 and TOX in A*02/S_269_- (green) specific and bulk (grey) CD8+ T cells. % expression among A*01/S_865_-, A*02/S_269_-and A*03/S_378_-specific non-naïve CD8+ T cells is shown on the right side. BL: baseline; dpp: days post prime; dpb: days post boost; GRZB: granzyme B. (d-e) Two-way ANOVA with main effects only comparing the effect of the different epitopes and of time course. All statistically significant results are marked with the respective exact p-value (p_e_: epitope, p_t_: time). Source data

**Extended Data Fig. 3:**
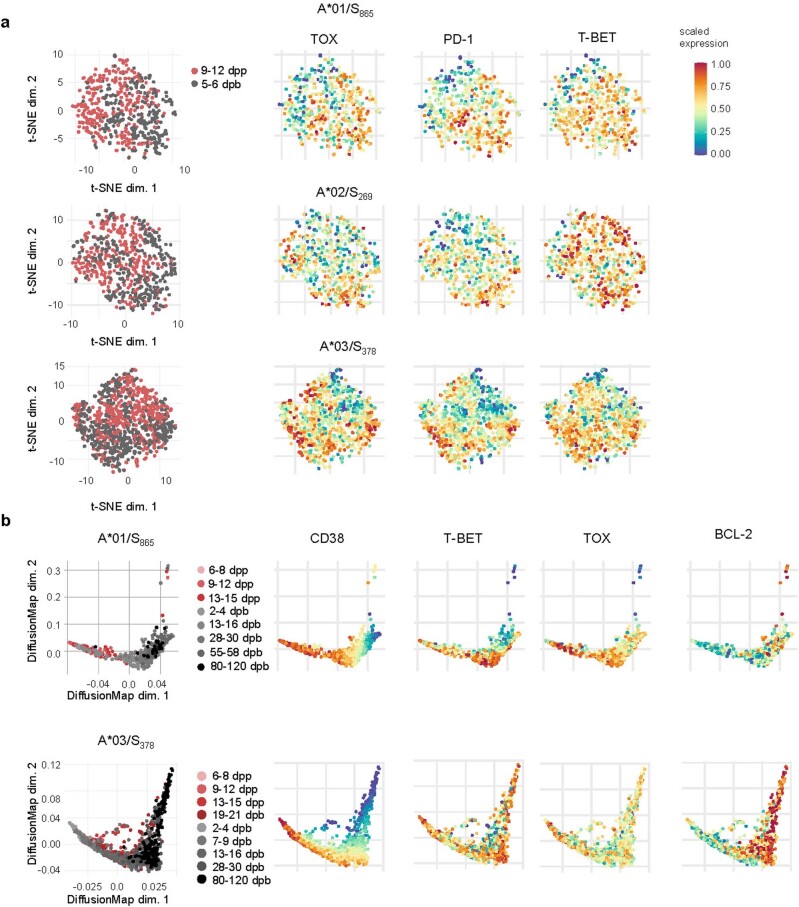
Expression levels of CD38, T-BET, TOX, PD-1 and BCL-2 after prime and boost vaccination. **(a)** t-SNE representation of flow cytometry data comparing A*01/S_865_-, A*02/S_269_- and A*03/S_378_-specific CD8+ T cells after prime and boost vaccination (prime 4, 3 and 5 and boost 3, 3 and 5 individuals for A*01/S_865_, A*02/S_269_ and A*03/S_378_, respectively). Expression levels of TOX, PD-1 and T-BET are indicated for A*01/S_865_, A*02/S_269_ and A*03/S_378_ (colour-code: blue, low expression; red, high expression). **(b)** Diffusion map showing flow cytometry data for A*01/S_865_- and A*03/S_378_-specific CD8+ T cells of one representative donor at dpp (shades of red) and dpb (shades of grey) with CD38, T-BET, TOX and BCL-2 expression levels plotted on the diffusion map (colour-code: blue, low expression; red, high expression). dpp: days post prime; dpb: days post boost; t-SNE: t-distributed stochastic neighbour embedding.

**Extended Data Fig. 4:**
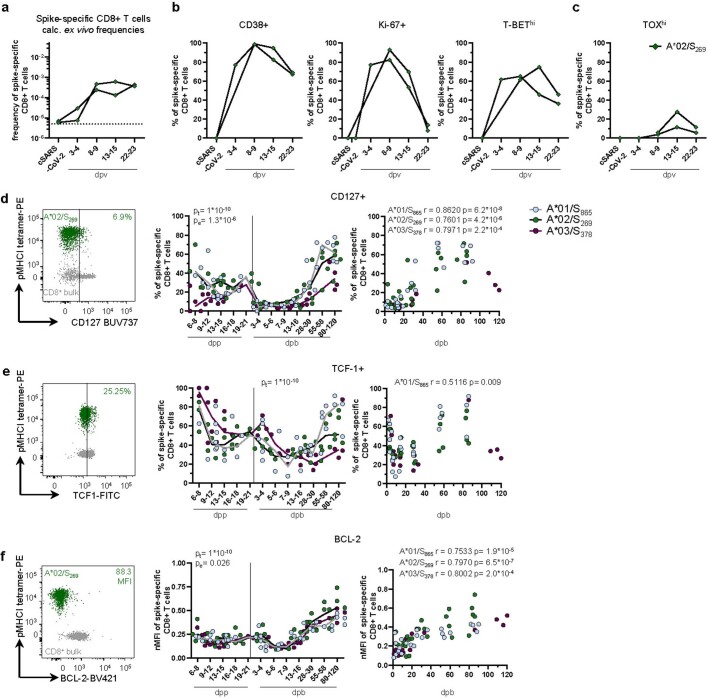
Spike-specific CD8+ T cells in SARS-CoV-2 convalescents after a single vaccination and expression of early memory marker after prime and boost vaccination. **(a-c)** Data of two donors who recovered from SARS-CoV-2 infection >365 day ago and received a single dose Bnt162b2 vaccination. **(a)** The calculated ex vivo frequency for A*02/S_269_-specific CD8+ T cells in two donors is indicated at baseline (cSARS-CoV-2) and days after single dose vaccination. % of CD38, Ki-67, T-BET^hi^ **(b)** and TOX^hi^ **(c)** expressing A*02/S_269_- specific non-naïve CD8+ T cells. **(d-e)** Exemplary dot plots depicting the expression levels of CD127 **(d)** and TCF-1 **(e)** in A*02/S_269_- (green) specific and bulk (grey) CD8+ T cells 6 dpb. % A*01/S_865_-, A*02/S_269_- and A*03/S_378_-specific non-naïve CD8+ T cells expressing CD127 **(d)** and TCF-1 **(e)** were determined. Correlation of indicated marker with dpb is depicted on the right. **(f)** Exemplary dot plots depicting the expression levels of BCL-2 in A*02/S_269_- (green) specific as well as on bulk (grey) CD8+ T cells 6 dpb. nMFI (MFI normalized to naïve CD8+ T cells) of A*01/S_865_-, A*02/S_269_- and A*03/S_378_-specific non-naïve CD8+ T cells expressing BCL-2 was determined. Correlation of nMFI BCL-2 with dpb is depicted on the right. cSARS: convalescent SARS; dpv: days post vaccination; BL: baseline; dpp: days post prime; dpb: days post boost; nMFI: normalized Median Fluorescent Intensity, epi: epitope. (d-f) Two-way ANOVA with main effects only comparing the effect of the different epitopes and of time course (left) and Spearman correlation (right) was performed. All statistically significant results are marked with the respective exact p-value (p_e_: epitope, p_t_: time). Source data

We longitudinally collected peripheral blood mononuclear cells (PBMCs) and sera in 3–4-day intervals from 32 healthcare workers (Supplementary Table 1) that had not been previously infected with SARS-CoV-2, starting before prime until day 80–120 after boost (Extended Data Fig. 1a) and analysed the induction of spike-specific CD8^+^ T cells that target A*01/S_865_, A*02/S_269_ and A*03/S_378_ epitopes in 4–5 individuals each (Extended Data Fig. 1b). All three epitopes are not highly conserved between SARS-CoV-2 and SARS-CoV-1, MERS or common cold coronaviruses (Extended Data Fig. 1c). Thus, the detected spike-specific CD8^+^ T cells indeed reflect a response to vaccination. The epitopes are not affected by the sequence variations present in the variants of concern (VOC) alpha, beta, gamma and delta (Extended Data Fig. 1c). The tested A*01-, A*02- and A*03-restricted CD8^+^ T cells that are part of a broader spike-specific CD8^+^ T cell response, however, proved to be dominant when analysing responses that span the whole S protein (Extended Data Fig. 1d). Ex vivo frequencies of A*01/S_865_-, A*02/S_269_- and A*03/S_378_-specific CD8^+^ T cells were rather low after vaccination (Extended Data Fig. 2a). To increase the detection rate and to allow subsequent comprehensive profiling, we performed pMHCI-tetramer enrichment (Extended Data Fig. 2b). We detected a rapid and substantial induction of spike-specific CD8^+^ T cells that were present in 9 out of 13 tested donors already at days 6–8 and peaked in most donors 9–12 days post prime (dpp) (Fig. 1a). The strong CD8^+^ T cell activation was also reflected by high expression of CD38 and Ki-67 as early as days 6–8 in most cells (Fig. 1b, c and Extended Data Fig. 2c). Boost vaccination led to a further increase of CD8^+^ T cell frequencies that peaked 5–6 days post boost (dpb) with a subsequent slow contraction phase that reached nearly pre-boost frequencies at about 80–120 dpb (Fig. 1a). Post-boost and post-prime expansion were accompanied by effector T (T_eff_) cell differentiation (high expression of Ki-67, CD38, granzyme B, PD-1, CD39, T-BET and TOX) (Fig. 1b and Extended Data Fig. 2c–e). However, *t*-distributed stochastic neighbour embedding (*t*-SNE) analysis revealed that CD8^+^ T_eff_ cells are qualitatively different at the peak expansion after boost (obtained at 5–6 dpb) compared with prime (obtained at 9–12 dpp) with a more consolidated cytotoxic effector cell phenotype (increased expression of T-BET, TOX and CD39) post boost (Extended Data Fig. 3a). This consolidated post-boost T_eff_ cell response is further supported by diffusion map analysis (Fig. 1c and Extended Data Fig. 3b). Specifically, diffusion map embedding revealed a continuous relationship of the longitudinally collected spike-specific CD8^+^ T cells after prime (depicted in reddish colours)/boost (depicted in grey colours) indicating a directed trajectory of the T_eff_ cell response. Along the trajectory, CD8^+^ T cells exhibited the highest expression of PD-1, TOX, T-BET and CD38 after boost indicating profound activation and progressing differentiation (Fig. 1c and Extended Data Fig. 3b). Of note, a single vaccine dose also induced boost expansion and strong activation but lower TOX expression (Extended Data Fig. 4a–c) of spike-specific CD8^+^ T cells in individuals who recovered from mild to moderate infection approximately 12 months before vaccination (Supplementary Table 1).

We also assessed the induction of spike-specific memory precursor CD8^+^ T cells that are characterized by CD127, BCL-2 and TCF-1 expression and are relevant for maintaining the CD8^+^ T cell response^13,14^. Roughly 20–30% of spike-specific CD8^+^ T cells expressed CD127 after prime followed by a transient reduction and subsequent strong increase after boost (Fig. 1d and Extended Data Fig. 4d). Expression dynamics of TCF-1 (Fig. 1e and Extended Data Fig. 4e) and BCL-2 (Extended Data Fig. 4f) were similar to CD127. However, the overall frequency of CD127^+^ (Fig. 1d) and TCF-1^+^ (Fig. 1e) spike-specific CD8^+^ T cells remained constant indicating a stable memory precursor pool induced already early after prime vaccination. Together, bnt162b2 vaccination vigorously induces a lasting spike-specific CD8^+^ T cell response rapidly after prime vaccination.

## CD8^+^ T cell function after vaccination

**Fig. 2:**
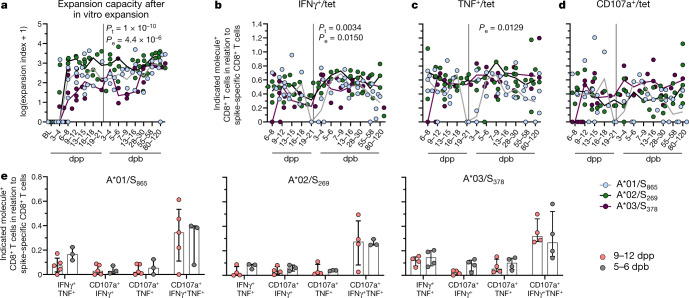
Functional capacities of vaccine-elicited spike-specific CD8^+^ T cells. **a**, Expansion capacity of spike-specific CD8^+^ T cells after in vitro expansion. **b**–**d**, Percentage of CD8^+^ T cells producing effector molecules related to the frequency of spike-specific CD8^+^ T cells. **e**, Bar graphs depicting the polyfunctionality of spike-specific CD8^+^ T cells comparing 9–12 dpp and 5–6 dpb vaccination. Line indicates median. Bar charts show the median with interquartile range (IQR). *P* values determined by two-way ANOVA with main effects only comparing the effect of the different epitopes and of time course (**a**–**d**) or by Mann–Whitney test with Holm–Šídák method (**e**). Source data

**Extended Data Fig. 5:**
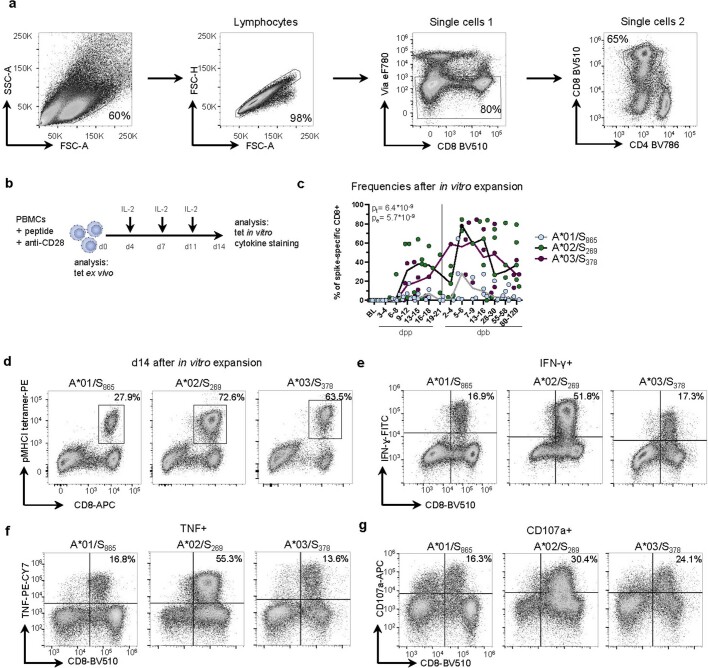
Frequency and functional capacity of spike-specific CD8+ T cells following prime and boost vaccination. **(a**) Gating strategy of flow cytometry data. Cytokine secretion of A*01/S_865_-, A*02/S_269_- and A*03/S_378_-specific CD8+ T cells was determined after in vitro expansion. **(b)** Workflow depicting peptide-specific in vitro expansion of CD8+ T cells. **(c)** Frequency of A*01/S_865_-, A*02/S_269_- and A*03/S_378_ -specific CD8+ T cells after 14 day of in vitro expansion. **(d-g)** Dot plots showing A*01/S_865_-, A*02/S_269_- and A*03/S_378_ -specific CD8+ T cells after in vitro expansion **(d)** and IFN-γ- **(e)**, TNF- **(f)** and CD107a- **(g)** producing CD8+ T cells after in vitro expansion (5-6 dpb). (c) Two-way ANOVA with main effects only comparing the effect of the different epitopes and of time course. BL: baseline; dpp: days post prime; dpb: days post boost, epi: epitope. Two-way ANOVA with main effects only comparing the effect of the different epitopes and of time course. All statistically significant results are marked with the respective exact p-value (p_e_: epitope, p_t_: time). Source data

After two weeks of peptide-specific in vitro expansion (Extended Data Fig. 5a, b), we detected higher frequencies of spike-specific CD8^+^ T cells after boost compared to prime vaccination (Extended Data Fig. 5c, d). However, the expansion index, a measure taking the input number of virus-specific CD8^+^ T cells into account was comparable for spike-specific CD8^+^ T cells after prime and boost vaccination, but differed between the A*01/S_865_- A*02/S_269_- and A*03/S_378_-specific CD8^+^ T cell responses (Fig. 2a). Thus, the increased frequencies of spike-specific CD8^+^ T cells after peptide-specific expansion most probably result from the increased ex vivo frequencies after boost. We also assessed spike-specific production of IFNγ and TNF (Extended Data Fig. 5e, f) and degranulation as indicated by CD107a expression (Extended Data Fig. 5g) in relation to the frequency of spike-specific CD8^+^ T cells after expansion as a measure of the effector function per cell. We observed reasonable effector capacity of circulating spike-specific CD8+ T cells obtained as early as 6–8 dpp (Fig. 2b–d). Similar to the expansion capacity, cytokine production and degranulation capacity remained nearly stable after boost compared to prime (Fig. 2b–e). Hence, functionally competent spike-specific CD8^+^ T cells that target different epitopes are substantially induced early after prime, and subsequent boost vaccination does not further increase their functional capacities in vitro.

## CD4^+^ T cells, B cells and antibodies

**Fig. 3:**
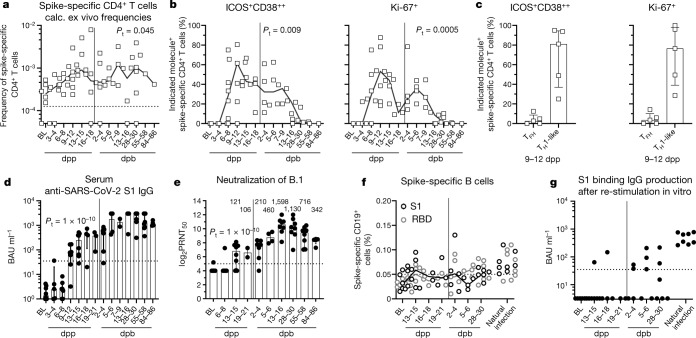
Circulating spike-specific CD4^+^ T cells, B cells and antibodies. **a**, Calculated ex vivo frequency of DRB1*15:01/S_236_-specific CD4^+^ T cells ex vivo after pMHCII tetramer-based enrichment is indicated at baseline, dpp and dpb. Detection limit: 1.25 × 10^4^. **b**, ICOS^+^CD38^++^ and Ki-67 expression within non-naive, DRB1*15:01/S_236_-specific CD4^+^ T cells. **c**, ICOS^+^CD38^++^ and Ki-67-expressing non-naive DRB1*15:01/S_236_-specific CD4^+^ T cells on 9–12 dpp within T_FH_ (CXCR5^+^PD-1^+^) and T_H_1-like (CXCR5^−^CXCR3^+^) cells. **d**, Anti-SARS-CoV-2 spike IgG at baseline and after vaccination (<35.2 binding antibody units (BAU) per ml: negative, ≥35.2 BAU ml^−1^: positive; upper limit of quantification: 3,000 BAU ml^−1^). **e**, Antibody neutralization activity is depicted as 50% plaque reduction neutralization tests (PRNT_50_) at baseline, dpp and dpb vaccination for the SARS-CoV-2 variant B.1. Numbers indicate non-logarithmic median value. Detection limit: 5 log_2_PRNT_50_. **f**, Percentage spike-specific B cells depicted at baseline, dpp and dpb as well as in natural infection for S1 and RBD. Detection limit: 0.05%. **g**, Secreted anti-SARS-CoV-2 spike IgG from PBMCs after in vitro stimulation with CpG and IL-2 (<35.2 BAU ml^−1^: negative, ≥35.2 BAU ml^−1^: positive). Line indicates median. Bar charts show the median and IQR. *P* values determined by one-way ANOVA with a mixed effects model comparing the effect of the time course (**a**, **b**, **f**), a Wilcoxon test (**c**) or a two-way ANOVA with main effects only comparing the effect of the different epitopes and of time course (**g**). Source data

**Extended Data Fig. 6:**
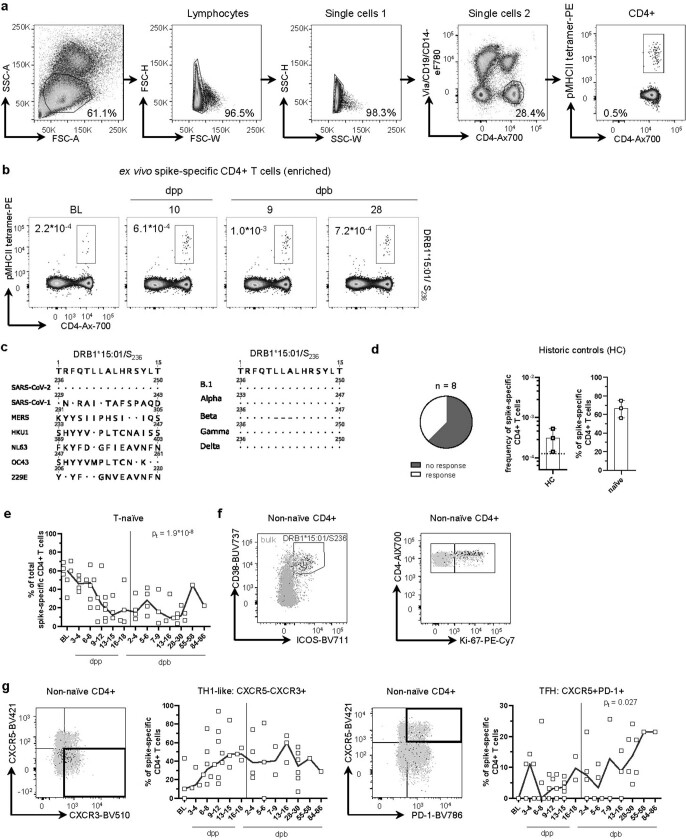
Circulating spike-specific CD4+ T cells following prime and boost vaccination. **(a)** Gating strategy of flow cytometry data. DRB1*15:01/S_236_-specific CD4+ T cells were identified via pMHCII tetramer-based enrichment used in further analyses. **(b)** Dot plots showing DRB1*15:01/S_236_-specific CD4+ T cells ex vivo after pMHCII tetramer-based enrichment at BL, before and after boost vaccination. **(c)** Comparison of DRB1*15:01/S_236_ epitope sequence with amino acid sequences of SARS-CoV-1/2, MERS and common cold coronaviruses (left) and of circulating SARS-CoV-2 variants of concern (VOC) (right). **(d)** Number of responses (left) and the calculated ex vivo frequencies (middle) of DRB1*15:01/S_236_-specific CD4+ T cells in historic controls (HC, *n* = 8). % naïve within total DRB1*15:01/S_236_-specific CD4+ T cells in historic controls (right). Detection limit: 1.25*10^−4^. **(e)** % naïve of total DRB1*15:01/S_236_-specific CD4+ T cells. **(f)** Representative dot plots of ICOS+CD38++ and Ki-67+ expression (grey: bulk, black: DRB1*15:01/S_236_-specific CD4+ T cells). **(g)** % CXCR5-CXCR3+ TH1 cells and of CXCR5+PD-1+ TFH within non-naïve DRB1*15:01/S_236_-specific CD4+ T cells with representative dot plots (grey: bulk, black: DRB1*15:01/S_236_-specific CD4+ T cells). BL: baseline, dpp: days post prime, dpb: days post boost; TFH: follicular helper T cells; TH1: T helper cells 1-like cells. Bar charts show the median with IQR. (e, g) One-way ANOVA with a mixed effects model comparing the effect of the time course. All statistically significant results are marked with the respective exact p-value (p_t_: time). Source data

Next, we longitudinally assessed circulating spike-specific CD4^+^ T cells that target DRB1*15:01/S_236_ (Extended Data Fig. 6a) after prime and boost vaccination in eight individuals (Supplementary Table 1). The selected DRB1*15:01/S_236_ epitope is unique for SARS-CoV-2 in comparison to SARS-CoV-1, MERS or common cold coronaviruses and conserved in circulating SARS-CoV-2 variants (B.1, alpha, gamma and delta) except for VOC beta (Extended Data Fig. 6c). The frequencies of DRB1*15:01/S_236_-specific CD4^+^ T cells were lower than CD8^+^ T cell responses but detectable after pMHCII tetramer-based enrichment (Extended Data Fig. 6b). At baseline and in historic control samples (banked before August 2019), spike-specific CD4^+^ T cells were detectable with a primarily naive phenotype (Extended Data Fig. 6d, e), which reflects the presence of antigen-unexperienced precursors. After vaccination, the proportion of naive spike-specific CD4^+^ T cells decreased, which suggests vaccine-induced activation (Extended Data Fig. 6e). However, compared with CD8^+^ T cells, we observed a lower mobilization of circulating spike-specific CD4^+^ T cells indicated by a limited increase of frequencies (Fig. 3a) and a smaller percentage of activated ICOS^+^CD38^++^ or Ki-67^+^ subsets (Fig. 3b and Extended Data Fig. 6f). Most activated DRB1*15:01/S_236_-specific CD4^+^ T cells exhibited a T_H_1 cell phenotype (Fig. 3c). In line with this observation, vaccine-induced spike-specific CD4^+^ T cells displayed a T_H_1 cell rather than a follicular helper T (T_FH_) cell phenotype (Extended Data Fig. 6g).

**Extended Data Fig. 7:**
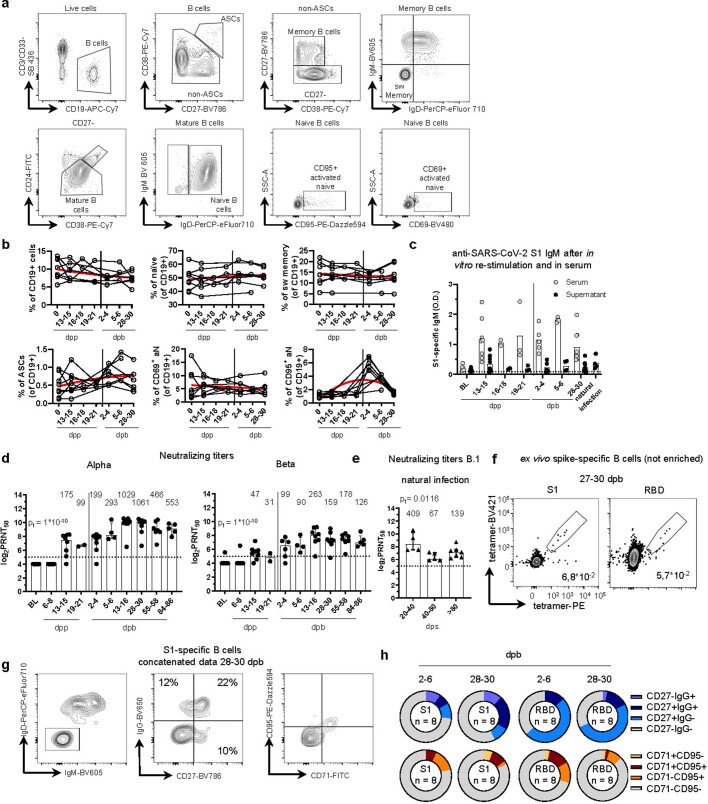
Circulating B cells, antibodies and antibody neutralization activity after prime and boost vaccination. **(a)** Gating strategy of flow cytometry data for different B cell subpopulations. **(b)** % of CD19+, naïve, switched memory, ASCs, CD69+aN and CD95+ aN cells within bulk B cells was determined at BL, post prime/boost. Nonlinear fit was calculated in red. **(c)** Detection of spike IgM in serum at BL, post prime/boost by ELISA. Level of secreted IgM was determined in supernatant of PBMC from BL, post prime/boost and after natural infection after in vitro stimulation for 9 d with CpG and IL-2. Detection limit: O.D. 8.5*10^−2^. **(d)** Antibody neutralization activity is depicted as PRNT_50_ at BL, dpp and dpb vaccination for the SARS-CoV-2 VOC alpha and beta. **(e)** Antibody neutralization capacity is depicted as PRNT_50_ at different dps in natural SARS-CoV-2 infection with SARS-CoV-2 ancestral variant B.1. (d-e) Numbers indicate non-logarithmic median value. Detection limit: 5 log_2_PRNT_50_. **(f)** Dot plots showing double tetramer positive B cells for S1 and RBD epitope at 28-30 dpb. **(g)** Dot plots representing co-expression of IgD/IgM (left), IgG/CD27 (middle) and CD95/CD71 (right) on concatenated flies of S1-specific B cells at 28-30 dpb. **(h)** Donut plots representing co-expression of CD27/IgG (upper) and CD71/CD95 (lower) in concatenated analysis of S1-specific B cells populations (n indicated the number of individual files concatenated) after boost vaccination. sw memory: switched memory; aN: activated naïve; BL: baseline; dpp: days post prime; dpb: days post boost; dps: days post symptoms; O.D.: optical density; PRNT_50_: plaque-reduction neutralization titer 50. Bar charts show the median with IQR. (c-e) One-way ANOVA with a mixed effects model comparing the effect the time course. All statistically significant results are marked with the respective exact p-value (p_t_:_:_ time). Source data

We then assessed the kinetics of the vaccine-induced humoral response. The distribution of peripheral B cell subpopulations was stable throughout prime or boost vaccination, with the exception of a progressively slight increase in antibody-secreting cells (ASC) (Extended Data Fig. 7a, b). An increase in the frequency of CD95^+^ B cells was observed shortly after boost, which indicates ongoing B cell activation via CD40-mediated T cell help and/or B cell receptor activation within secondary lymphoid organs^15^ (Extended Data Fig. 7b). In line with the appearance of activated B cells in the periphery, we observed a progressive maturation of the serum antibody response with S1-specific IgM present after prime whereas S1-specific IgG reasonably detectable after boost (Fig. 3d and Extended Data Fig. 7c), coinciding with a high neutralization capacity in SARS-CoV-2 plaque reduction assays. More precisely, SARS-CoV-2 B.1 and VOC alpha were similarly well neutralized by post-boost sera, whereas the cross-neutralization activity against VOC beta was reduced approximately by a factor of 5 (Fig. 3e and Extended Data Fig. 7d). Neutralization capacity of post-boost sera was clearly increased compared with time point-matched mild infection (Extended Data Fig. 7e). In line with the progressive maturation of the antibody response, S1- and receptor-binding domain (RBD)-specific B cells (Extended Data Fig. 7f) largely remained below the ex vivo detection limit until the first week post boost (Fig. 3f). The delayed appearance of circulating S1-specific B cells was confirmed by polyclonal restimulation in vitro (Fig. 3g), which showed a limited presence of class-switched B cells that could produce S1-specific IgG before boost. S1-specific B cells were largely unswitched after prime (Extended Data Fig. 7g, h), also reflected by S1-specific IgM production upon polyclonal restimulation in vitro (Extended Data Fig. 7c), and acquired a memory phenotype after boost vaccination (Extended Data Fig. 7g, h). In addition, after boost vaccination, S1-specific B cells showed increased transferrin receptor (CD71) and CD95 expression (Extended Data Fig. 7g, h), which indicates their germinal centre origin^16^. Hence, bnt162b2 vaccination efficiently elicits a protective humoral immune response, composed of ASC and antigen-specific memory B cells that are mobilized to the periphery after boost.

## Early memory CD8^+^ T cells

**Fig. 4:**
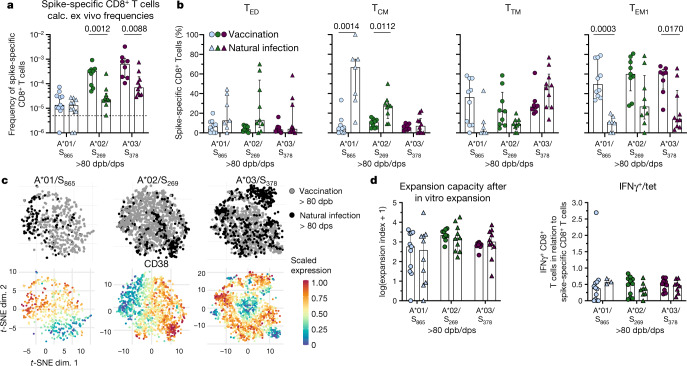
Early memory CD8^+^ T cells after vaccination and natural infection. **a**, Calculated frequency of spike-specific CD8^+^ T cells 80–200 dpb vaccination or dps in natural infection. Detection limit: 5 × 10^−6^. **b**, Distribution of spike-specific CD8^+^ T cell memory subsets 80–200 dpb/dps. **c**, *t*-SNE representation of flow cytometry data, depicting spike-specific CD8^+^ T cells more than 80 dpb vaccination and dps of natural infection (grey: vaccination, black: natural infection) for A*01/S_865_- (vaccination *n* = 9, natural infection *n* = 9), A*02/S_269_-(vaccination *n* = 10, natural infection *n* = 8) and A*03/S_378_- (vaccination *n* = 9, natural infection *n* = 9) specific CD8^+^ T cells. **d**, Left, expansion index of spike-specific CD8^+^ T cells after in vitro expansion at 80-200 dpb/dps. Right, percentage of IFNγ-producing CD8^+^ T cells related to the frequency of spike-specific CD8^+^ T cells after in vitro expansion at 80–200 dpb/dps. T_TM_, transitional memory T cells. Bar charts show the median with IQR. *P* values were determined by Mann–Whitney test with Holm–Šídák method. Source data

**Extended Data Fig. 8:**
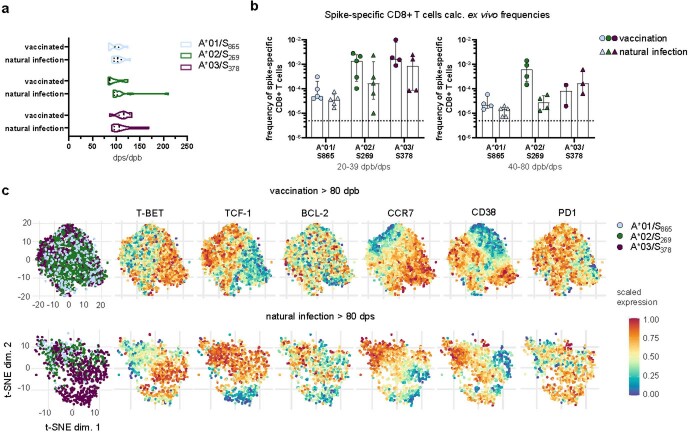
Comparison of spike-specific CD8+ T cells after vaccination and natural infection. **(a**) Distribution of dpb and dps of donors analysed for A*01/S_865_-, A*02/S_269_- and A*03/S_378_-specific CD8+ T cells after vaccination and natural infection. **(b)** Calculated frequency of A*01/S_865_-, A*02/S_269_- and A*03/S_378_-specific CD8+ T cells ex vivo after pMHCI tetramer-based enrichment at 20-39 and 40-80 dpb/dps. Detection limit: 5*10^−6^. **(c)** Plotted expression levels of T-BET, TCF-1, BCL-2, CCR7, CD38 and PD1 on t-SNE depicting all spike-specific CD8+ T cells >80 dpb vaccination (upper) and >80 dps in natural SARS-CoV-2 infection (lower) are indicated for A*01/S_865_, A*02/S_269_ and A*03/S_378_ (colour-code: blue, low expression; red, high expression). dpb: days post boost; dps: days post symptom onset; t-SNE: t-distributed stochastic neighbour embedding. Bar charts show the median with IQR. (a) 2-way ANOVA including Tukey’s multiple comparisons test were performed. (b) Statistical analyses of vaccination vs. natural infection was performed by Mann–Whitney test with Holm-Šídák method. All statistically significant results are marked with the respective exact p-value. Source data

**Extended Data Fig. 9:**
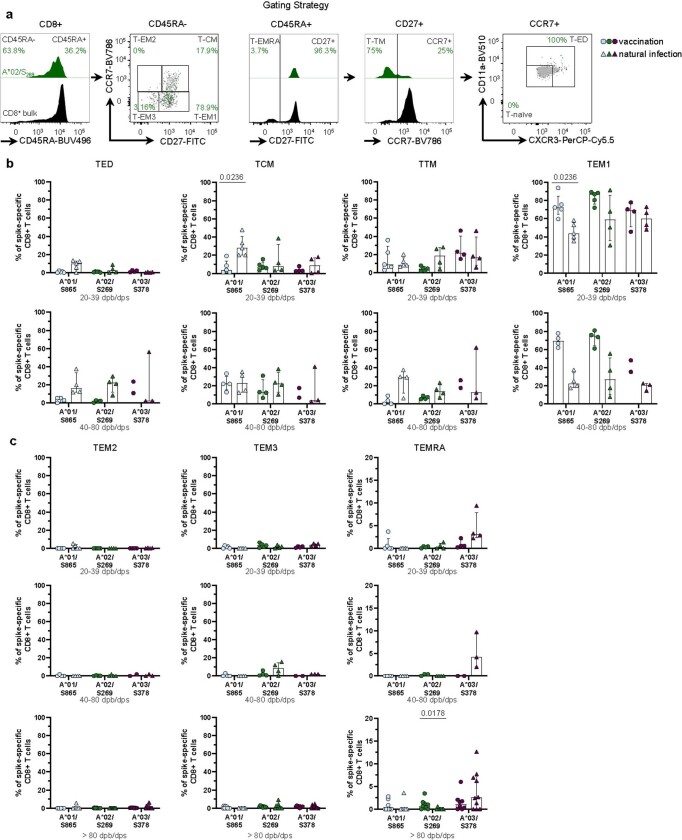
Subset distribution of spike-specific CD8+ T cell after vaccination and natural infection. **(a)** Gating strategy of memory CD8+ T cell populations among A*02/S_269_- (green) (36 dps) specific CD8+ T cells, (grey/black: bulk CD8+ T cells). **(b)** Distribution of spike-specific CD8+ T-cell memory subsets TED, TCM, TTM and TEM1 at 20-39 and 40-80 dpb/dps. **(c)** Distribution of spike-specific CD8+ T-cell memory subsets TEM2, TEM3 and TEMRA at 20-39, 40-80 and >80 dpb/dps. dpb: days post boost; dps: days post symptoms; ; TED: early differentiated, TCM: central memory T cells and TEM1: effector memory T cells 1 ; TEM2: effector memory T cells 2; TEM3: effector memory T cells 3, TEMRA terminally differentiated effector memory cells re‐expressing CD45RA. Bar charts show the median with IQR. (b-c) Statistical analyses of vaccination vs. natural infection was performed by Mann–Whitney test with Holm-Šídák method. All statistically significant results are marked with the respective exact p-value. Source data

**Extended Data Fig. 10:**
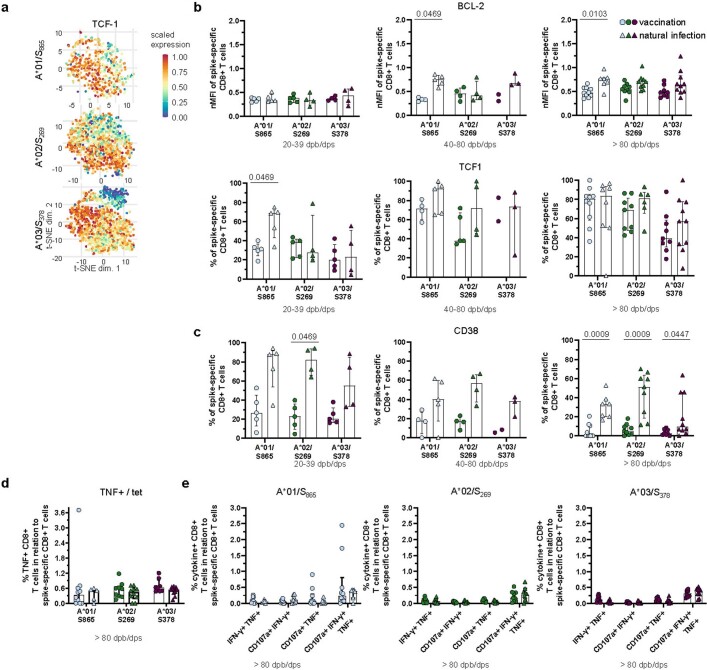
Expression levels of BCL-2, TCF1 and CD38 and polyfunctionality of spike-specific CD8+ T cells after vaccination and natural infection. **(a)** Plotted expression levels of TCF-1 on t-SNE depicting spike-specific CD8+ T cells >80 dpb vaccination and dps of natural infection for A*01/S_865_- (vaccination *n* = 9, natural infection *n* = 9), A*02/S_269_-(vaccination *n* = 10, natural infection *n* = 8) and A*03/S_378_- (vaccination *n* = 9, natural infection *n* = 9) (colour-code: blue, low expression; red, high expression). **(b, c)** nMFI (normalized to naïve CD8+ T cells) of BCL-2 (b, upper), TCF1 (b, middle) and CD38 (c, lower) spike-specific non-naïve CD8+ T cells at 20-39, 40-80 and >80 dpb/dps. **(d)** % of TNF-producing CD8+ T cells in relation to the frequency of spike-specific CD8+ T cells after in vitro expansion. **(e)** Bar graphs depicting the polyfunctionality of the respective spike-specific CD8+ T cells comparing vaccination and natural infection. dpb: days post boost; dps: days post symptom onset. Bar charts show the median with IQR. (b-e) Statistical analyses of vaccination vs. natural infection was performed by Mann–Whitney test with Holm-Šídák method. All statistically significant results are marked with the respective exact p-value. Source data

We compared vaccine-elicited spike-specific early memory CD8^+^ T cells (days post boost vaccination) with time point-matched T cells induced by natural infection (days post symptom onset) (Extended Data Fig. 8a). A*01/S_865_-specific CD8^+^ T cell frequencies were similar after vaccination versus infection at all time points analysed. However, in comparison to vaccination, lower frequencies of A*02/S_269_- and A*03/S_378_-specific CD8^+^ T cells were detectable at days 80–120 (6 out of 30 (natural infected), 4 out of 28 (vaccinees) were obtained at days 120–200) after natural infection (Fig. 4a and Extended Data Fig. 8b). Phenotypic characteristics of early memory CD8^+^ T cells targeting A*01/S_865_, A*02/S_269_ and A*03/S_378_ differed after vaccination versus natural infection as revealed by *t*-SNE analyses (Extended Data Fig. 8c). Possible reasons for this include differences in their MHCI binding and presentation characteristics (Extended Data Fig. 1c). In addition, we also observed differences in T cell memory subset distribution (Fig. 4b and Extended Data Fig. 9a–c) of spike-specific early memory CD8^+^ T cells with higher fractions of more early differentiated subsets, for example, early differentiated (T_ED_) and central memory (T_CM_) T cells for A*01/S_865_- and A*02/S_269_-specific CD8^+^ T cells and transitional memory cells for A*03/S_378_-specific CD8^+^ T cells after natural infection (80–120 dps). By contrast, higher frequencies of effector memory 1 T cells (T_EM1_) were detectable after vaccination (80–120 dpb) (Fig. 4b). Spike-specific effector memory 2 and 3 T cells (T_EM2_ and T_EM3_) and terminally differentiated effector memory T cells that expressed CD45RA (T_EMRA_) were hardly detectable in the circulation (Extended Data Fig. 9c). Of note, the memory subset distribution of A*03/S_378_-specific CD8^+^ T cells differed from A*01/S_865_- and A*02/S_269_-specific CD8^+^ T cells with only a minor fraction of T_ED_ and T_CM_ cells targeting A*03/S_378_ reflecting an overall further differentiation towards effector memory subsets (Fig. 4b and Extended Data Fig. 9b). *t*-SNE analysis of concatenated expression data further supports qualitative differences of spike-specific CD8^+^ T cells obtained from the early memory phase (80–120 dpb/dps) after vaccination compared to natural infection being less pronounced for A*03/S_378_-specific CD8^+^ T cells (Fig. 4c). For A*01/S_865_-specific CD8^+^ T cells we also observed higher expression of TCF-1 and BCL-2 after natural infection (Extended Data Fig. 10a, b). Both *t*-SNE analysis and manual gating demonstrated a higher and prolonged CD38 expression on spike-specific CD8^+^ T cells after natural infection (Fig. 4c and Extended Data Fig. 10c). However, vaccine- and natural infection-associated expansion capacity and cytokine production of spike-specific CD8^+^ T cells were similar (Fig. 4d and Extended Data Fig. 10d, e). Hence, compared with natural infection, vaccine-associated spike-specific early memory CD8^+^ T cell populations exhibit similar functional capacities but a different subset distribution.

## Discussion

In summary, a robust, stable and fully functional spike-specific CD8^+^ T cell response is elicited already after prime vaccination at a time point when neutralizing antibodies were hardly detectable and coincides with the protective effect observed for mRNA vaccines that starts at 10–12 dpp^2,3^. In contrast to CD8^+^ T cells, peak mobilization of neutralizing antibodies and antigen-specific B cells to the periphery was first detectable after boost. This is in line with previous reports^4,7,11,12^ and most probably represents maturation of the response in secondary lymphoid organs^17^ with subsequent release to the circulation. After boost, highly cross-neutralizing antibodies are present in the sera, clearly adding a major protective effector mechanism on top of the early-mobilized spike-specific CD8^+^ T cell response. The humoral and CD8^+^ T cell response are potentially coordinated by early elicited spike-reactive CD4^+^ T cells^8^ that underwent a limited boost expansion after second dose mRNA vaccination supporting their coordinating role.

Fully functional vaccine-elicited early memory CD8^+^ T cells patrol the periphery for SARS-CoV-2 at least within the first months. The functional capacity of spike-specific early memory CD8^+^ T cells is similar after vaccination and natural infection up to 3–4 months after boost or symptom onset. Compared with natural infection, however, the early memory pool of spike-specific CD8^+^ T cells after vaccination exhibits a different memory T cell subset distribution that may affect long-term maintenance characteristics^18^. This difference may be caused by differential duration and location of antigen contact and different inflammatory responses after vaccination versus infection^19,20^, as indicated by a lower CD38 expression on early memory spike-specific CD8^+^ T cells after vaccination compared with natural infection^4,21^. Follow-up studies including larger cohorts of vaccinees and SARS-CoV-2 convalescent individuals are clearly required to assess longevity of CD8^+^ T cell immunity. Our study was limited to circulating spike-specific adaptive immunity, and did not address local immunity at the viral entry site, the respiratory tract. However, our data provide insights into the protective mechanisms that underlie bnt162b2 vaccination with implications for the development of vaccination strategies against emerging pathogens and cancer.

## Methods

### Study cohort

In total, 32 healthcare workers that received a prime and boost vaccination with the mRNA vaccine bnt162b2/Comirnaty, 59 convalescent individuals following a mild course of SARS-CoV-2 infection, 2 convalescent individuals given one dose of bnt162b2/Comirnaty 12 months after infection, and historic controls (sampled before August 2019) of 8 healthy individuals were recruited at the Freiburg University Medical Center, Germany. A mild course of infection was characterized by clinical symptoms without respiratory insufficiency. SARS-CoV-2 infection was confirmed by positive PCR testing from oropharyngeal swab and/or SARS-CoV-2 spike IgG positive antibody testing. Donor characteristics are summarized in Supplementary Table 1. HLA-typing was performed by next-generation sequencing and is listed in Supplementary Table 1.

### Ethics

Written informed consent was obtained from all participants and the study was conducted according to federal guidelines, local ethics committee regulations (Albert-Ludwigs-Universität, Freiburg, Germany; vote: 322/20, 21-1135 and 315/20) and the Declaration of Helsinki (1975).

### PBMC isolation

Venous blood samples were collected in EDTA-anticoagulated tubes. PBMCs were isolated with lymphocyte separation medium density gradients (Pancoll separation medium, PAN Biotech GmbH) and stored at −80 °C. Frozen PBMCs were thawed in complete medium (RPMI 1640 supplemented with 10% fetal calf serum, 1% penicillin/streptomycin and 1.5% HEPES buffer 1 M (all additives from Thermo Scientific) containing 50 U ml^−1^ benzonase (Sigma).

### Sequence alignment

Sequence homology analyses were performed in Geneious Prime 2020.0.3 (https://www.geneious.com/) using Clustal Omega 1.2.2 alignment with default settings^22^. Reference genomes of human coronaviruses 229E (NC_002645), HKU1 (NC_006577), NL63 (NC_005831), OC43 (NC_006213), MERS (NC_019843), SARS-CoV-1 (NC_004718) and SARS-CoV-2 (MN908947.3) were downloaded from NCBI database. Spike proteins of human coronaviruses were aligned according to their homology (amino acid level). Analysed spike SARS-CoV-2 epitopes were then mapped to the corresponding protein alignment. Correspondingly mutation analyses were performed with the spike protein of VOC alpha, beta, gamma and delta.

### In vitro expansion and intracellular IFNγ staining with overlapping peptides

A total of 182 overlapping peptides that spanned the SARS-CoV-2 spike sequence (Gene Bank Accession code MN908947.3) were synthesized as 18-mers overlapping by 11 amino acids with a free amine NH_2_ terminus and a free acid COOH terminus with standard Fmoc chemistry and a purity of >70% (Genaxxon Bioscience). In vitro expansion with OLPs was performed as follows: 20% of the PBMCs were stimulated with a pool of all 181 SARS-CoV-2 spike OLPs (10 μg ml^−1^) for 1 h at 37 °C, washed and co-cultured with the remaining PBMCs in RPMI medium supplemented 20 U ml^−1^ with recombinant IL-2. On day 10, intracellular IFNγ staining was performed with pooled OLPs (45 pools with 4 OLP each). Therefore, cells were re-stimulated with OLP pools (50 μM), DMSO as negative control or PMA and ionomycin as positive control in the presence of brefeldin A an IL-2. After 5 h of incubation at 37 °C, cells were stained for surface markers (CD8^+^, CD4^+^; Viaprobe) and intracellular markers (IFNγ). Subsequently, on day 12 the single overlapping peptides of positive pools were tested by intracellular cytokine staining. Viral amino acid sequences of positive individual OLPs were analysed for pre-described minimal epitopes or the best HLA-matched predicted candidate using the Immune Epitope Database website (using two prediction algorithms ANN 4.0 and NetMHCpan EL 4.1^23^ for 8-mer, 9-mer and 10-mer peptides with half-maximal inhibitory concentration (IC_50_) of <500 nM).

### Peptides and tetramers for T cell analysis

Peptides were synthesized with an unmodified N terminus and an amidated C terminus with standard Fmoc chemistry and a purity of >70% (Genaxxon Bioscience). Peptide was loaded on HLA class I easYmers (immunAware) according to manufacturer’s instructions (A*01/S_865_ LTDEMIAQY, A*02/S_269_ YLQPRTFLL and A*03/S_378_ KCYGVSPTK). SARS-CoV-2 peptide-loaded HLA class I tetramers were produced by conjugation of biotinylated peptide-loaded HLA class I easYmers with phycoerythrin (PE)-conjugated streptavidin (Agilent) according to the manufacturer’s instructions. A SARS-CoV-2-specific HLA class II custom tetramer (DRB1*15:01/S_236_ TRFQTLLALHRSYLT) was obtained from (MBL).

### In vitro expansion of spike-specific CD8^+^ T cells and assessment of effector function

Approximately 1.5 × 10^6^ PBMCs were stimulated with A*01/S_865_, A*02/S_269_ or A*03/S_378_-specific peptides (5 μM) and anti-CD28 monoclonal antibody (0.5 μg ml^−1^, BD) and expanded for 14 days in complete RPMI culture medium containing rIL-2 (20 IU ml^−1^, StemCell Technologies). Intracellular cytokine production and degranulation was assessed with spike-specific peptides (15 μM) in the presence of anti-CD107a (H4A3, 1:100) (BD Bioscience) for 1 h at 37 °C. Afterwards, brefeldin A (GolgiPlug, 0.5 μl ml^−1^) and monensin (GolgiStop, 0.5 μl ml^−1^) (all BD Biosciences) were added for additional 5 h, followed by surface and intracellular staining. The expansion capacity was calculated based on peptide-loaded HLA class I tetramer staining as previously described^24^.

### Magnetic bead-based enrichment of spike-specific CD8^+^ T cells

Spike-specific CD8^+^ T cells were enriched as previously described^25^. In brief, 1 × 10^7^–2 × 10^7^ PBMCs (with an average of 15.7% CD8^+^ T cells) were labelled with PE-coupled peptide-loaded HLA class I tetramers for 30 min. Enrichment was then performed using anti-PE beads with MACS technology (Miltenyi Biotec) according to the manufacturer’s instructions. Subsequently, enriched spike-specific CD8^+^ T cells were analysed by multiparametric flow cytometry and frequencies of spike-specific CD8^+^ T cells were calculated as described before^25^. Only enriched samples with ≥5 spike-specific CD8 T cells were included in further analyses, resulting in a detection limit of 5 × 10^−6^.

### Magnetic bead-based enrichment of spike-specific CD4^+^ T cells

Enrichment of spike-specific CD4^+^ T cells was adapted from the method described previously^25^. In brief, 1.5 × 10^7^–2 × 10^7^ PBMCs of DRB1*15:01-positive donors were labelled with PE-coupled peptide-loaded MHC class II tetramers for 40 min. Then, 5 μl was taken from 1,000 μl pre-enriched sample (1:200) and used for subsequent flow cytometric staining. Subsequent enrichment was performed with anti-PE beads using MACS technology (Miltenyi Biotec) according to the manufacturer’s protocol. Enriched spike-specific CD4^+^ T cells and the pre-enriched sample were used for flow cytometric staining. The complete pre-enriched and enriched samples were recorded. Only enriched samples with ≥5 spike-specific CD4^+^ T cells were included in further analyses. The frequency of spike-specific CD4^+^ T cells was calculated as follows: Absolute number of spike-specific CD4^+^ T cells (enriched sample) divided by the absolute number of CD4^+^ T cells (pre-enriched sample) × 200. The detection limit as a frequency was calculated as follows: 5 spike-specific CD4^+^ T cells (enriched sample) divided by the mean number of CD4^+^ T cells (pre-enriched sample) throughout all tested donors × 200.

### Multiparametric flow cytometry for T cell analysis

The following antibodies were used for multiparametric flow cytometry: anti-CCR7-PE-CF594 (150503, 1:50), anti-CCR7-BUV395 (3D12, 1:25), anti-CD4-BV786 (L200, 1:200), anti-CD8-BUV395 (RPA-T8, 1:400), anti-CD8-BUV510 (SK1, 1:100), anti-CD8-APC (SK-1, 1:200), anti-CD11a-BV510 (HI111, 1:25), anti-CD28-BV421 (CD28.2, 1:100), anti-CD38-APC-R700 (HIT2, 1:400), anti-CD38-BUV737 (HB7, 1:200), anti-CD39-BV650 (TU66, 33:1), anti-CD45RA-BUV496 (HI100, 1:800), anti-CD45RA-BUV737 (HI100, 1:200), anti-CD69-BUV395 (FN50, 1:50), anti-CD107a-APC (H4A3, 1:100), anti-CD127-BUV737 (HIL-7R-M21, 1:50), anti-CD127-BV421 (HIL-7R-M21, 3:100), anti-EOMES-PerCP-eF710 (WD1928, 1:50), anti-Granzyme B-PE-CF594 (GB11, 1:100), anti-ICOS-BV711 (DX29, 1:100), anti-IFN-γ-FITC (25723.11, 1:8), anti-IL-21-PE (3A3-N2.1, 1:25), anti-PD-1-BV605 (EH12.1, 1:50), anti-PD-1-PE-Cy7 (EH12.2H7, 1:200), anti-PD-1-BV786 (EH12.1, 1013122, 3:100), anti-T-BET-PE-CF594 (O4-46,93533305, 3:100), anti-TNF-PE-Cy7 (Mab11, 1:400) (BD Biosciences), anti-BCL-2-BV421 (100, 1:200), anti-CCR7-BV785 (G043H7, 1:50), anti-CD4-AlexaFluor700 (RPA-T4, 300526, 1:200), anti-CD25-BV650 (BC96, 1:33), anti-CD57-BV605 (QA17A04, 1:100), anti-CD127-BV605 (A019D5, 3:100), anti-CXCR3-PerCP-Cy5.5 (G025H7, 1:33), anti-CXCR3-BV510 (G025H7, 3:100), anti-CXCR5-BV421 (J252D4, 1:100), anti-IL-2-PerCP-Cy5.5 (MQ1-17H12, 1:100), anti-Ki-67-BV711 (Ki-67, 1:200), anti-Ki-67-PE-Cy7 (Ki-67, 1:200) (BioLegend), anti-TCF-1-AlexaFluor488 (C63D9, 1:100) (Cell Signaling), anti-CD14-APC-eFluor780 (61D3, 1:400), anti-CD19-APC-eFluor780 (HIB19, 1:400), anti-CD27-FITC (0323, 1:100), anti-KLRG1-BV711 (13F12F2, 1:50), anti-T-BET-PE-Cy7 (4B10, 1:200), anti-TOX-eFluor660 (TRX10, 1:100) (Thermo Fisher), anti-CD45RA-PerCP-Cy5.5 (HI100, 3:100) (Invitrogen). For live/dead discrimination a fixable Viability Dye (APC-eFluor780 1:200, 1:400) (Thermo Fisher) or ViaProbe (7-AAD, 1:33) (BD Biosciences)) was used. FoxP3/Transcription Factor Staining Buffer Set (Thermo Fisher) and Fixation/Permeabilization Solution Kit (BD Biosciences) were used according to the manufacturer’s protocol to stain for intranuclear and cytoplasmic molecules, respectively. After fixation of cells in 2% paraformaldehyde (PFA, Sigma), analyses were performed on FACSCanto II, LSRFortessa with FACSDiva software version 10.6.2 (BD) or CytoFLEX (Beckman Coulter) with CytExpert Software version 2.3.0.84. Data were analysed with FlowJo 10.6.2 (Treestar).

### Dimensional reduction of multiparametric flow cytometry data

Dimensionality reduction of multiparametric flow cytometry data was done with R version 4.0.2 using the Bioconductor (release (3.11)) CATALYST package23. The analyses were performed on gated virus-specific CD8+ T cells including the markers CD69, CD45RA, BCL-2, PD1, CD25, Ki-67, TCF-1, EOMES, CCR7, T-BET, TOX and CD38. Downsampling of cells to 100 or 200 cells (*t*-SNE or diffusion maps) was performed before dimensionality reduction to facilitate the visualization of different samples. Marker intensities were transformed by arcsinh (inverse hyperbolic sine) with a cofactor of 150. Dimensionality reduction on the transformed data was achieved by *t*-SNE and diffusion map visualization.

### S1- and RBD-tetramerization for B cell analysis

A biotinylated form of recombinant S1 and RBD proteins (BioLegend) were tetramerized by addition of PE-conjugated or BV421-conjugated streptavidin (BioLegend) and used for B cell tetramer staining assays. In brief, streptavidin-PE or streptavidin-BV21 was added in an amount that equals one-fifth of the monomer substrate amount. The streptavidin was added in five equal portions to the monomer and incubated each time at 4 °C for 20 min on a shaker. The tetramers were filled up to 100 μl with 0.1% BSA in PBS and stored at 4 °C.

### Multiparametric flow cytometry for B cell analysis

Phenotype of vaccinated individuals’ PBMCs was determined by flow cytometry with the following antibodies: anti-CD20-BV510 (2H7, 1:80), anti-IgM-BV605 (MHM-88, 1:200), anti-CD71-FITC (CY1G4, 1:1000), anti-CD95-PE-Dazzle594 (DX2, 1:50), anti-CD24-FITC (ML5, 1:100), anti-CD38-PE-Cy7 (HB-7, 1:300), anti-BAFF-R-AF647 (11C1, 1:100), anti-CD19-APC-Cy7 (HIB19, 1:150) (BioLegend); anti-IgG-BV650 (G18-145, 1:600), anti-CD27-BV786 (L128, 1:100), anti-CD69-BV480 (FN50, 1:200) (BD Biosciences); anti-IgA-PerCP (polyclonal, 1:200) (Jackson ImmunoResearch); anti-CD3-SB-436 (OKT3, 1:200), anti-CD33-Super Bright 436 (WM-53, 1:50), anti-IgD-PerCP-eFluor 710 (IA6-2, 1:200) (Invitrogen). Dead cell exclusion was performed by Zombie NIR Fixable Viability Kit (Biolegend, 1:800). Multiparametric flow cytometry data was collected on Cytek Aurora with SpectroFlo Software version 2.2.0.3.

### In vitro PBMCs activation and ELISA

PBMCs of vaccinated individuals and patients with a history of SARS-CoV-2 infection were plated at 0.5 × 10^6^ cells ml^−1^ and polyclonally stimulated for 9 days with thiol-modified CpG (0.25 μM, TCGTCGTTTTGTCGTTTTGTCGTT) and hIL-2 (100 ng/ml, Immunotools). At day 9, the supernatants of the in vitro culture were cleared from debris by centrifugation and used to determine the presence of SARS-CoV-2 spike-specific IgG antibodies (Anti-SARS-CoV-2-QuantiVac-ELISA (IgG), Euroimmun) according to the manufacturer’s instructions. To detect S1 specific IgM, supernatant of the in vitro culture and serum of vaccinated individuals was incubated on a S1 pre-coated plate (Anti-SARS-COV-2, Euroimmun). Bound IgM was detected with alkaline phosphatase-conjugated anti-human IgM (Jackson ImmunoResearch), and developed with p-nitrophenyl phosphate (Sigma-Aldrich) in DEA buffer.

### Serum IgG determination

SARS-CoV-2-specific antibodies were determined by Anti-SARS-CoV-2-QuantiVac-ELISA (IgG) from Euroimmun detecting anti-SARS-CoV-2 spike IgG (anti-SARS-CoV-2 S IgG; <35.2 BAU ml^−1^: negative, ≥ 35.2 BAU ml^−1^: positive) according to the manufacturer’s instructions.

### Neutralization assay

Samples of vaccinated and convalescent individuals were tested in a plaque reduction neutralization assay. In brief, VeroE6 cells were seeded in 12-well plates at a density of 2.8 × 10^5^ cells per well 24 h before infection. Serum samples were diluted at ratios of 1:16, 1:32, 1:64, 1:128, 1:256, 1:512 and 1:1,024 in 50 μl PBS total volume. For each sample, one negative control was included (PBS without serum). Diluted sera and negative controls were subsequently mixed with 90 plaque-forming units (PFU) of authentic SARS-CoV-2 (either B.1, alpha or beta variant) in 50 μl PBS (1,600 PFU ml^−1^) resulting in final sera dilution ratios of 1:32, 1:64, 1:128, 1:256, 1:512, 1:1,024 and 1:2,048. After incubation at room temperature for 1 h, 400 μl PBS was added to each sample and the mixture was subsequently used to infect VeroE6 cells. After 1.5 h of incubation at room temperature, inoculum was removed and the cells were overlaid with 0.6% Oxoid-agar in DMEM, 20 mM HEPES (pH 7.4), 0.1% NaHCO_3_, 1% BSA and 0.01% DEAE-Dextran. Cells were fixed 72 h after infection using 4% formaldehyde for 30 min and stained with 1% crystal violet upon removal of the agar overlay. PFU were counted manually. Plaques counted for serum-treated wells were compared to the average number of plaques in the untreated negative controls, which were set to 100%. The PRNT_50_ value was calculated using a linear regression model in GraphPad Prism 9 (GraphPad Prism Software).

### Statistics

Statistical analysis was performed with GraphPad Prism 9 (GraphPad Prism Software). Statistical significance was assessed by one-way ANOVA with a mixed effects model, two-way ANOVA with main effects only, two-tailed Mann–Whitney test with Holm–Šídák multiple comparison, Wilcoxon test and Spearman correlation. Analyses were performed in independent experiments. Statistics was performed for Figs. 1a, b, d, e, 2, Extended Data Figs. 2d, e, 4d, e, f, 5c in *n* = 5 longitudinally analysed vaccines for A*01/S_865_ and A*02/S_269_ and *n* = 4 longitudinally analysed vaccines for A*03/S_378_; for Fig. 3a, b, c, Extended Data Fig. 6e, g in *n* = 8 longitudinally analysed vaccinees for DRB1*15:01/S_236_; for Fig. 3d, e in 8 longitudinally analysed vaccines; for Fig. 3f, g, Extended Data Fig. 7c in *n* = 8 longitudinally analysed vaccines and *n* = 8 donors with a history of natural SARS-CoV-2 infection cross-sectionally; for Fig. 4a, b, d, Extended Data Fig. 10 in *n* = 11 cross-sectionally analysed vaccinees for A*01/S_865_ at 80–120 dpb, *n* = 9 cross-sectionally analysed vaccinees for A*02/S_269_ at 80-120 dpb, *n* = 8 cross-sectionally analysed vaccinees for A*03/S_378_ at 80–120 dpb, *n* = 10 donors with a history of natural SARS-CoV-2 infection cross-sectionally for A*01/S_865_ 80–120 dpb, *n* = 10 donors with a history of natural SARS-CoV-2 infection cross-sectionally for A*02/S_269_ 80–120 dpb and *n* = 10 donors with a history of natural SARS-CoV-2 infection cross-sectionally for A*03/S_378_ 80-120 dpb; Extended Data Fig. 7b, h in *n* = 8 longitudinally analysed vaccines; Extended Data Fig. 7d in *n* = 7 longitudinally analysed vaccinees; for Extended Data Fig. 7e in *n* = 16 donors with a history of natural SARS-CoV-2 infection cross-sectionally/longitudinally; for Extended Data Fig. 8b, 9b, c in *n* = 5 longitudinally analysed vaccinees for A*01/S865 (*n* = 5 at 20–40 dpb and 4 at 40–80 dpb), *n* = 5 longitudinally analysed vaccinees for A*02/S269 (*n* = 5 at 20–40 dpb and 4 at 40–80 dpb), *n* = 4 longitudinally analysed vaccinees for A*03/S378 (*n* = 4 at 20–40 dpb and 2 at 40–80 dpb), *n* = 9 donors with a history of natural SARS-CoV-2 infection cross-sectionally for A*01/S865 (*n* = 5 at 20–40 dpb, 4 at 40–80 dpb), *n* = 8 donors with a history of natural SARS-CoV-2 infection cross-sectionally for A*02/S269 (*n* = 4 at 20–40 dpb, 4 at 40–80 dpb), *n* = 7 donors with a history of natural SARS-CoV-2 infection cross-sectionally for A*03/S378 (*n* = 4 at 20–40 dpb, 3 at 40–80 dpb). *P < 0.05; **P < 0.01; ***P < 0.001; ****P < 0.0001.

### Reporting summary

Further information on research design is available in the Nature Research Reporting Summary linked to this paper.

## Online content

Any methods, additional references, Nature Research reporting summaries, source data, extended data, supplementary information, acknowledgements, peer review information; details of author contributions and competing interests; and statements of data and code availability are available at 10.1038/s41586-021-03841-4.

## Supplementary information

Supplementary Table 1 This table lists donor characteristics.

Reporting Summary

Peer Review File

## Data Availability

Patient-related data not included in the paper were generated as part of clinical examination and may be subject to patient confidentiality. Further raw and supporting data conflicting with patient confidentl, are available from the corresponding authors upon request (response within two weeks). Requests for these data will be reviewed by the corresponding authors to verify if the request is subject to any intellectual property or confidentiality obligations. Reference viral sequences SARS-CoV-2 (MN908947.3) https://www.ncbi.nlm.nih.gov/nuccore/MN908947, 229E (NC_002645) https://www.ncbi.nlm.nih.gov/nuccore/NC_002645, HKU1 (NC_006577) https://www.ncbi.nlm.nih.gov/nuccore/NC_006577, NL63 (NC_005831) https://www.ncbi.nlm.nih.gov/nuccore/NC_005831, OC43 (NC_006213) https://www.ncbi.nlm.nih.gov/nuccore/NC_006213, MERS (NC_019843) https://www.ncbi.nlm.nih.gov/nuccore/NC_019843, SARS-CoV-1 (NC_004718) https://www.ncbi.nlm.nih.gov/nuccore/NC_004718) were downloaded from the NCBI database (https://www.ncbi.nlm.nih.gov/). Any data and materials that can be shared will be released via a Material Transfer Agreement. Source data are provided with this paper.

## Source data

Source Data Fig. 1
Source Data Fig. 2
Source Data Fig. 3
Source Data Fig. 4
Source Data Extended Data Fig. 1
Source Data Extended Data Fig. 2
Source Data Extended Data Fig. 4
Source Data Extended Data Fig. 5
Source Data Extended Data Fig. 6
Source Data Extended Data Fig. 7
Source Data Extended Data Fig. 8
Source Data Extended Data Fig. 9
Source Data Extended Data Fig. 10

## Code availability

R code to reproduce the analyses of multiparametric flow-cytometry data are available at https://github.com/sagar161286/SARSCoV2_specific_CD8_Tcells.
