# Supplementary material for: Rapid and stable mobilization of CD8+ T cells by SARS-CoV-2 mRNA vaccine
Source: Nature. 2021 Jul 28;597(7875):268–73. doi: 10.1038/s41586-021-03841-4 (PMC8426185; doi:10.1038/s41586-021-03841-4)
Supplement: Supplementary file 1 — This table lists donor characteristics. [file 41586_2021_3841_MOESM1_ESM.pdf]

nature portfolio

https://doi.org/10.1038/s41586-021-03841-4

Supplementary information

Rapid and stable mobilization of CD8 T cells by SARS-CoV-2 mRNA vaccine

In the format provided by the authors and unedited

Supplementary Information for

Rapid and stable mobilization of CD8+ T cells by SARS-CoV-2 mRNA vaccine

Valerie Oberhardt[1,2]*, Hendrik Luxenburger[1,3]* Janine Kemming[1,2]*, Isabel Schulien[1]*, Kevin Ciminski[4], Sebastian Giese[4], Benedikt Csernalabics[1], Julia Lang-Meli[1,3], Iga Janowska[5], Julian Staniek[2,5], Katharina Wild[1,6], Kristi Basho[1], Mircea Stefan Marinescu[1], Jonas Fuchs[4], Fernando Topfstedt[5], Ales Janda[7], Oezlem Sogukpinar[1], Hanna Hilger[1], Katarina Stete[1], Florian Emmerich[8], Bertram Bengsch[1,9], Cornelius F. Waller[10], Siegbert Rieg[1], Sagar[1], Tobias Boettler[1,11], Katharina Zoldan[1], Georg Kochs[4], Martin Schwemmle[4], Marta Rizzi[5], Robert Thimme[1#§], Christoph Neumann-Haefelin[1#§], Maike Hofmann[1#§]

*equally contributing first authors; #equally contributing last authors; §corresponding authors

Materials and Correspondence:

Maike Hofmann: maike.hofmann@uniklinik-freiburg.de

Christoph Neumann-Haefelin: christoph.neumann-haefelin@uniklinik-freiburg.de

Robert Thimme: robert.thimme@uniklinik-freiburg.de

Hugstetter Straße 55, 79106 Freiburg, Germany

This PDF includes:

Supplementary Table 1: Donor characteristics

Supplementary Table 1: Donor characteristics									
Donor ID	Sex	Age	HLA Type	Cohort	Natural infection	Tested Epitopes	Serum S1 IgG1 assay	Neutralizing Antibodies (Plaque reduction assay)	Overlapping peptides (OLP)
V1	f	45	A*02:01, A*33:03, B*44:03, B*58:01, DRB1*07:01	vaccinated	-	CD8 T cells: A*02/S269-277<br>B cells: S1, RBD	yes	yes	no
V2	f	44	A*01:01, A*11:01, B*15:17, B*35:01, DRB1*01:03, DRB1*13:02	vaccinated	-	CD8 T cells: A*01/S865-873<br>B cells: S1, RBD	yes	yes	yes
V3	f	30	A*01:01, A*02:01, B*07:02, B*37:01, DRB1*05:01, DRB1*11:03	vaccinated	-	CD8 T cells: A*01/S865-873, A*02/S269-277<br>B cells: S1, RBD	yes	yes	yes
V4	m	25	A*02:01, A*29:02, B*45:01, B*51:01, DRB1*04:01, DRB1*13:01	vaccinated	-	CD8 T cells: A*02/S269-277<br>B cells: S1, RBD	yes	yes	no
V5	m	47	A*01:01, A*11:01, B*08:01, B*15:01, DRB1*01:01, DRB1*03:01	vaccinated	-	CD8 T cells: A*01/S865-873<br>B cells: S1, RBD	yes	yes	no
V6	m	62	A*01:01, A*02:01, B*08:01, B*15:01, DRB1*08:01, DRB1*11:01	vaccinated	-	CD8 T cells: A*01/S865-873, A*02/S269-277<br>B cells: S1, RBD	yes	yes	no
V7 / HC 1	f	36	A*01:01, A*03:01, B*07:02, B*57:01, DRB1*07:01, DRB1*15:01	vaccinated / historic control	-	CD8 T cells: A*01/S865-873<br>CD4: DRB1*15:01/ S236-250<br>B cells: S1, RBD	yes	yes	yes
V8	m	31	A*02:01, B*15:01, B*51:01, DRB1*01:01, DRB1*11:01	vaccinated	-	CD8 T cells: A*02/S269-277<br>B cells: S1, RBD	yes	yes	no
V9	m	41	A*01:01, A*03:01, B*08:01, B*35:01, DRB1*01:01, DRB1*03:01	vaccinated	-	B cells: S1, RBD	no	yes	yes
V10	m	39	A*02:01, A*23:01, B*13:02, B*58:01, DRB1*07:01, DRB1*13:01	vaccinated	-	B cells: S1, RBD	no	yes	no
V11 / HC2	m	33	A*02:01, A*03:01, B*07:02, DRB1*15:01	vaccinated / historic control	-	CD8 T cells: A*03/S378-386<br>CD4 T cells: DRB1*15:01/S236-250	no	yes	yes
V12 / HC3	m	41	A*01:01, A*02:01, B*08:01, B*40:01, DRB1*14:02, DRB1*15:01	vaccinated / historic control	-	CD4 T cells: DRB1*15:01/ S236-250	no	yes	yes
V13	f	37	A*02:01, A*68:01, B*15:01, B*40:01, DRB1*13:01, DRB1*15:01	vaccinated	-	CD8 T cells: A*02/S269-277<br>CD4 T cells: DRB1*15:01/ S236-250	no	yes	yes
V14	m	35	A*01:01, A*26:01, B*07:02, B*27:05, DRB1*01:01, DRB1*15:01	vaccinated	-	CD8 T cells: A*01/S865-873<br>CD4 T cells: DRB1*15:01/ S236-250	no	yes	yes
V15	m	35	A*01:01, A*24:02, B*07:02, B*27:05, DRB1*15:01, DRB1*16:01	vaccinated	-	CD8 T cells: A*01/S865-873<br>CD4 T cells: DRB1*15:01/ S236-250	no	yes	no
V16/HC4	f	60	A*02:01, B*07:02, B*44:02, DRB1*04:01, DRB1*15:01	vaccinated / historic control	-	CD8 T cells: A*02/S269-277<br>CD4 T cells: DRB1*15:01/ S236-250	no	yes	no
V17	m	40	A*02:01, A*03:01, B*07:02, B*57:01, DRB1*07:01, DRB1*15:01	vaccinated	-	CD8 T cells: A*03/S378-386<br>CD4 T cells: DRB1*15:01/S236-250	no	yes	no
V18	m	30	A*02:01, A*03:01, B*15:01, B*57:03, DRB1*04:01, DRB1*14:01	vaccinated	-	-	no	no	yes
V19	m	43	A*03:01, A*32:01, B*07:02, B*40:02, DRB1*11:01, DRB1*14:54	vaccinated	-	CD8 T cells: A*03/S378-386	no	no	yes
V20	m	32	A*03:01, B*07:02, B*44:02, DRB1*11:119, DRB1*15:01	vaccinated	-	CD8 T cells: A*03/S378-386	no	no	yes
V21	m	31	A*01:01, A*03:01, B*08:01, B*18:01, DRB1*03:01, DRB1*13:01	vaccinated	-	CD8 T cells: A*03/S378-386	no	no	no
V22	m	30	A*01:01, A*02:01, B*13:02, B*35:02, DRB1*07:01, DRB1*11:02	vaccinated	-	CD8 T cells: A*01/S865-873, A*02/S269-277	no	no	yes
V23	f	50	A*03:01, B*07:02, B*27:05, DRB1*13:03, DRB1*15:01	vaccinated	-	CD8 T cells: A*03/S378-386	no	no	no
V24	f	31	A*01:01, B*57:01, DRB1*07:01, DRB1*11:01	vaccinated	-	CD8 T cells: A*01/S865-873	no	no	no
V25	f	50	A*01:01, A*24:02, B*08:01, B*27:05, DRB1*01:01, DRB1*07:01	vaccinated	-	CD8 T cells: A*01/S865-873	no	no	no
V26	f	36	A*02:01, A*68:01, B*15:01, B*55:01, DRB1*03:02, DRB1*04:01	vaccinated	-	CD8 T cells: A*02/S269-277	no	no	no
V27	f	35	A*03:01, A*25:01, B*07:02, B*18:01, DRB1*06:02, DRB1*15:01	vaccinated	-	CD8 T cells: A*03/S378-386	no	no	no
V28	m	31	A*03:01, A*30:01, B*13:02, B*35:01, DRB1*12:01, DRB1*15:01	vaccinated	-	CD8 T cells: A*03/S378-386	no	no	no
V29	f	30	A*26:01, B*07:02, B*38:01, DRB1*04:01, DRB1*15:01	vaccinated	-	-	no	no	yes
V30	m	47	A*02:01, A*24:02, B*27:05, B*51:01, DRB1*11:01, DRB1*11:04	vaccinated	-	-	no	no	yes
V31	m	60	A*02:01, B*08:01, B*15:01, DRB1*03:01, DRB1*11:01	vaccinated	-	-	no	no	yes
V32	f	51	A*02:01, A*68:01, B*15:01, B*44:02, DRB1*11:03, DRB1*14:54	vaccinated	-	-	no	no	yes
IV1	m	57	A*02:01, A*68:01, B*07:02, B*44:02, DRB1*11:01, DRB1*15:01	resolved 04/2020 / vaccinated 04/2021	mild infection	CD8 T cells: A*02/S269-277	no	no	no
IV2	f	56	A*02:01, A*31:01, B*07:02, B*15:01, DRB1*07:01, DRB1*15:01	resolved 04/2020 / vaccinated 04/2021	mild infection	CD8 T cells: A*02/S269-277	no	no	no
HC5	m	33	A*24:02, A*29:02, B*07:02, B*44:03, DRB1*14:54, DRB1*15:01	historic control	-	CD4 T cells: DRB1*15:01/ S236-250	no	no	no
HC6	m	33	A*02:01, A*03:01, B*07:02, DRB1*04:01, DRB1*15:01	historic control	-	CD4 T cells: DRB1*15:01/ S236-250	no	no	no
HC7	m	30	A*01:01, A*03:01, B*08:01, B*35:03, DRB1*03:01, DRB1*15:01	historic control	-	CD4 T cells: DRB1*15:01/ S236-250	no	no	no
HC8	f	35	A*02:01, A*26:01, B*07:02, B*38:01, DRB1*13:01, DRB1*15:01	historic control	-	CD4 T cells: DRB1*15:01/ S236-250	no	no	no
I1	m	34	A*03:01, A*24:02, B*44:03, B*45:01, DRB1*04:01, DRB1*15:01	resolved	mild infection	CD8 T cells: A*03/S378-386	no	no	no
I2	m	88	A*02:01, A*25:01, B*44:02, DRB1*01:01, DRB1*11:01	resolved	mild infection	CD8 T cells: A*02/S269-277	no	no	no
I3	f	86	A*02:01, A*31:01, B*13:02, B*40:01, DRB1*04:01, DRB1*15:01	acute resolving	mild infection	CD8 T cells: A*02/S269-277	yes	no	no
I4	m	63	A*02:01, B*44:02, B*58:01, DRB1*03:01, DRB1*13:01	resolved	mild infection	CD8 T cells: A*02/S269-277	yes	no	no
I5	f	38	A*01:01, A*11:01, B*51:01, B*52:01, DRB1*04:02, DRB1*15:06	resolved	mild infection	CD8 T cells: A*01/S865-873	no	no	no
I6	m	79	A*02:01, B*15:01, B*44:02, DRB1*03:01, DRB1*04:04	resolved	mild infection	CD8 T cells: A*02/S269-277	no	no	no
I7	m	69	A*01:01, A*25:01, B*08:01, B*18:01, DRB1*04:01, DRB1*15:01	resolved	mild infection	CD8 T cells: A*01/S865-873	yes	yes	no
I8	f	39	A*01:01, A*30:02, B*18:01, B*41:01, DRB1*07:01, DRB1*07:11	resolved	mild infection	CD8 T cells: A*01/S865-873	yes	yes	no
I9	m	34	A*01:01, A*03:01, B*08:01, DRB1*03:01	resolved	mild infection	CD8 T cells: A*01/S865-873	yes	yes	no
I10	m	57	A*02:01, A*24:02, B*44:02, B*57:01, DRB1*07:01, DRB1*13:01	resolved	mild infection	CD8 T cells: A*02/S269-277	yes	no	no
I11	m	37	A*01:01, A*29:02, B*07:02, B*40:01, DRB1*01:03, DRB1*11:01	resolved	mild infection	CD8 T cells: A*01/S865-873	yes	yes	no
I12	m	36	A*01:01, A*03:01, B*37:01, B*40:01, DRB1*13:02, DRB1*15:01	resolved	mild infection	CD8 T cells: A*01/S865-873	yes	yes	no
I13	f	46	A*02:01, A*11:01, B*07:02, B*49:01, DRB1*04:01, DRB1*13:02	resolved	mild infection	CD8 T cells: A*02/S269-277	no	no	no

I14	m	30	A*01:01, A*02:01, B*15:01, B*51:01, DRB1*11:01, DRB1*13:01	resolved	mild infection	CD8 T cells: A*01/S865-873, A*02/S269-277	yes	yes	no
I15	m	29	A*01:01, A*31:01, B*39:01, B*51:01, DRB1*01:01, DRB1*12:01	resolved	mild infection	CD8 T cells: A*01/S865-873	yes	no	no
I16	m	56	A*01:01, A*03:01, B*07:02, B*08:01, DRB1*03:01, DRB1*15:01	resolved	mild infection	CD8 T cells: A*01/S865-873	yes	yes	no
I17	m	38	A*02:01, A*31:01, B*07:02, B*15:01, DRB1*12:01, DRB1*15:01	resolved	mild infection	CD8 T cells: A*02/S269-277	yes	no	no
I18	m	27	A*02:01, A*31:01, B*07:02, B*15:01, DRB1*12:01, DRB1*15:01	resolved	mild infection	CD8 T cells: A*02/S269-277	yes	no	no
I19	m	22	A*02:01, B*15:01, B*40:01, DRB1*08:01, DRB1*13:02	resolved	mild infection	CD8 T cells: A*02/S269-277	yes	no	no
I20	f	43	A*02:01, A*31:01, B*27:02, B*39:01, DRB1*12:01, DRB1*16:01	resolved	mild infection	CD8 T cells: A*02/S269-277	yes	no	no
I21	f	32	A*02:01, B*18:01, B*38:01, DRB1*07:01, DRB1*13:01	resolved	mild infection	CD8 T cells: A*02/S269-277	yes	no	no
I22	f	42	A*01:01, A*32:01, B*07:02, B*40:02, DRB1*07:01, DRB1*13:01	resolved	mild infection	CD8 T cells: A*01/S865-873	yes	yes	no
I23	f	32	A*02:01, A*03:01, B*40:01, B*44:02, DRB1*11:04, DRB1*13:01	resolved	mild infection	CD8 T cells: A*02/S269-277	yes	yes	no
I24	f	62	A*02:01, A*32:01, B*27:05, B*51:01, DRB1*11:01, DRB1*15:01	resolved	mild infection	CD8 T cells: A*02/S269-277	yes	yes	no
I25	f	48	A*02:01, A*03:01, B*07:02, B*13:02, DRB1*07:01, DRB1*13:05	resolved	mild infection	CD8 T cells: A*02/S269-277	yes	yes	no
I26	f	36	A*01:01, A*03:01, B*07:02, B*08:01, DRB1*03:01, DRB1*04:01	resolved	mild infection	CD8 T cells: A*01/S865-873	yes	no	no
I27	f	48	-	resolved	mild infection	B cells: S1, RBD	no	no	no
I28	m	50	-	resolved	mild infection	B cells: S1, RBD	no	no	no
I29	m	21	-	resolved	asymptomatic	B cells: S1, RBD	no	no	no
I30	m	58	-	resolved	asymptomatic	B cells: S1, RBD	no	no	no
I31	f	41	-	resolved	mild infection	B cells: S1, RBD	no	no	no
I32	m	42	-	resolved	mild infection	B cells: S1, RBD	no	no	no
I33	f	42	-	resolved	mild infection	B cells: S1, RBD	no	no	no
I34	m	41	-	resolved	mild infection	B cells: S1, RBD	no	no	no
I35	f	34	-	resolved	mild infection	B cells: S1, RBD	no	no	no
I36	m	35	-	resolved	mild infection	B cells: S1, RBD	no	no	no
I37	f	73	A*02:01, B*15:01, B*40:01, DRB1*04:04, DRB1*16:05	resolved	mild infection	CD8 T cells: A*02/S269-277	no	no	no
I38	m	77	A*01:01, A*11:01, B*13:02, B*39:01, DRB1*07:01, DRB1*16:01	resolved	mild infection	CD8 T cells: A*01/S865-873	no	no	no
I39	f	41	A*03:01, A*11:01, B*15:01, B*35:01, DRB1*04:07, DRB1*13:01	resolved	mild infection	CD8 T cells: A*03/S378-386	no	no	no
I40	f	34	A*03:01, A*26:08, B*15:01, B*35:03, DRB1*07:01, DRB1*11:01	resolved	mild infection	CD8 T cells: A*03/S378-386	no	no	no
I41	m	52	A*03:01, A*24:02, B*08:01, B*39:06, DRB1*03:01	resolved	mild infection	CD8 T cells: A*03/S378-386	no	no	no
I42	m	30	A*03:01, A*24:02, B*07:02, B*44:02, DRB1*01:01, DRB1*03:01	resolved	mild infection	CD8 T cells: A*03/S378-386	no	no	no
I43	f	46	A*03:01, A*34:02, B*07:02, B*35:03, DRB1*04:03, DRB1*15:01	resolved	mild infection	CD8 T cells: A*03/S378-386	no	no	no
I44	f	72	A*03:01, B*35:01, B*40:02, DRB1*04:03, DRB1*15:01	resolved	mild infection	CD8 T cells: A*03/S378-386	no	no	no
I45	m	55	A*03:01, B*07:02, B*35:01, DRB1*01:01, DRB1*15:01	resolved	mild infection	CD8 T cells: A*03/S378-386	no	no	no
I46	m	54	A*03:01, A*11:01, B*07:02, B*08:01, DRB1*03:01, DRB1*15:01	resolved	mild infection	CD8 T cells: A*03/S378-386	no	no	no
I47	m	52	A*03:01, A*30:01, B*07:02, B*50:01, DRB1*04:03, DRB1*14:54	resolved	mild infection	CD8 T cells: A*03/S378-386	no	no	no
I48	m	52	A*01:01, A*03:01, B*07:02, B*08:01, DRB1*03:01, DRB1*15:01	resolved	mild infection	CD8 T cells: A*01/S865-873	no	no	no
I49	f	43	A*01:01, A*68:01, B*08:01, B*44:02, DRB1*03:01, DRB1*07:01	resolved	mild infection	CD8 T cells: A*01/S865-873	no	no	no
I50	f	54	A*02:01, A*03:01, B*15:01, B*18:01, DRB1*04:01, DRB1*11:04	resolved	mild infection	CD8 T cells: A*02/S269-277	no	no	no
I51	f	44	A*02:01, A*03:01, B*07:02, B*35:08, DRB1*01:01, DRB1*07:01	resolved	mild infection	CD8 T cells: A*02/S269-277	no	no	no
I52	m	49	A*01:01, A*02:01, B*35:02, B*57:01, DRB1*07:01, DRB1*08:01	resolved	mild infection	CD8 T cells: A*01/S865-873, A*02/S269-277	no	no	no
I53	m	56	A*01:01, A*03:01, B*08:01, B*35:01, DRB1*11:01, DRB1*13:02	resolved	mild infection	CD8 T cells: A*01/S865-873, A*03/S378-386	no	no	no
I54	f	59	A*01:01, A*02:01, B*08:01, B*15:01, DRB1*01:01, DRB1*03:01	resolved	mild infection	CD8 T cells: A*01/S865-873, A*02/S269-277	no	no	no
I55	f	39	A*03:01, A*25:01, B*07:02, B*44:02, DRB1*11:01, DRB1*15:01	resolved	mild infection	CD8 T cells: A*03/S378-386	no	no	no
I56	f	63	A*03:01, A*25:01, B*07:02, B*18:01, DRB1*01:01, DRB1*15:01	resolved	mild infection	CD8 T cells: A*03/S378-386	no	no	no
I57	f	59	A*03:01, A*32:01, B*35:01, DRB1*04:02, DRB1*11:03	resolved	mild infection	CD8 T cells: A*03/S378-386	no	no	no
I58	f	29	A*01:01, A*32:01, B*08:01, B*44:02, DRB1*01:01, DRB1*03:01	resolved	mild infection	CD8 T cells: A*01/S865-873	no	no	no
I59	m	39	A*01:01, A*02:01, B*08:01, B*52:01, DRB1*03:01, DRB1*15:02	resolved	mild infection	CD8 T cells: A*01/S865-873, A*02/S269-277	no	no	no
