# Supplementary material for: Rapid and stable mobilization of CD8+ T cells by SARS-CoV-2 mRNA vaccine
Source: Nature. 2021 Jul 28;597(7875):268–73. doi: 10.1038/s41586-021-03841-4 (PMC8426185; doi:10.1038/s41586-021-03841-4)
Supplement: Supplementary file 2 — Reporting Summary [file 41586_2021_3841_MOESM2_ESM.pdf]

nature research

Corresponding author(s): Robert Thimme
Maike Hofmann
Christoph Neumann-Haefelin

Last updated by author(s): Jun 28, 2021

Reporting Summary

Nature Research wishes to improve the reproducibility of the work that we publish. This form provides structure for consistency and transparency in reporting. For further information on Nature Research policies, see our Editorial Policies and the Editorial Policy Checklist.

Statistics

For all statistical analyses, confirm that the following items are present in the figure legend, table legend, main text, or Methods section.

n/a	Confirmed	
☐	☒	The exact sample size (*n*) for each experimental group/condition, given as a discrete number and unit of measurement
☐	☒	A statement on whether measurements were taken from distinct samples or whether the same sample was measured repeatedly
☐	☒	The statistical test(s) used AND whether they are one- or two-sided<br>*Only common tests should be described solely by name; describe more complex techniques in the Methods section.*
☐	☒	A description of all covariates tested
☐	☒	A description of any assumptions or corrections, such as tests of normality and adjustment for multiple comparisons
☐	☒	A full description of the statistical parameters including central tendency (e.g. means) or other basic estimates (e.g. regression coefficient) AND variation (e.g. standard deviation) or associated estimates of uncertainty (e.g. confidence intervals)
☐	☒	For null hypothesis testing, the test statistic (e.g. *F*, *t*, *r*) with confidence intervals, effect sizes, degrees of freedom and *P* value noted<br>*Give P values as exact values whenever suitable.*
☐	☒	For Bayesian analysis, information on the choice of priors and Markov chain Monte Carlo settings
☐	☒	For hierarchical and complex designs, identification of the appropriate level for tests and full reporting of outcomes
☐	☒	Estimates of effect sizes (e.g. Cohen's *d*, Pearson's *r*), indicating how they were calculated

Our web collection on statistics for biologists contains articles on many of the points above.

Software and code

Policy information about availability of computer code

Data collection	All software used to perform data collection are described in the methods section of the manuscript or the supportive information. Multiparametric Flow cytometry data was collected on FACSCanto II, LSRFortessa with FACSDiva software version 10.6.2 (BD, Germany) or CytoFLEX (Beckman Coulter) with CytExpert Software version 2.3.0.84 or Cytek Aurora (Cytek Biosciences) with SpectroFlo® Software version 2.2.0.3. ELISA data was collected by SparkControl magellan software version 2.2.
Data analysis	All codes used to perform bioinformatic analyses are described in the methods section of the manuscript or the supportive information. Multiparametric flow cytometry data was analyzed using FlowJo software version 10.6.2 (Treestar, Becton Dickinson). The visualization of multiparametric flow cytometry data was done with R version 4.0.2 using the Bioconductor (version: Release (3.11)) CATALYST package (Crowell H, Zanotelli V, Chevrier S, Robinson M (2020). CATALYST: Cytometry dATa anALYSis Tools. R package version 1.12.2, https://github.com/HelenaLC/CATALYST). R code to reproduce the analyses of multiparametric flow-cytometry data is available at https://github.com/sagar161286/SARSCoV2_specific_CD8_Tcells. Visualization and statistical analysis was performed using GraphPad 9 software. Sequence homology analyses were performed in Geneious Prime 2020.0.3 (https://www.geneious.com/) using Clustal Omega 1.2.2 alignment with default settings. Reference viral sequences SARS-CoV-2 (MN908947.3) https://www.ncbi.nlm.nih.gov/nuccore/MN908947, 229E (NC_002645) https://www.ncbi.nlm.nih.gov/nuccore/NC_002645, HKU1 (NC_006577) https://www.ncbi.nlm.nih.gov/nuccore/NC_006577, NL63 (NC_005831) https://www.ncbi.nlm.nih.gov/nuccore/NC_005831, OC43 (NC_006213) https://www.ncbi.nlm.nih.gov/nuccore/NC_006213, MERS (NC_019843) https://www.ncbi.nlm.nih.gov/nuccore/NC_019843, SARS-CoV-1 (NC_004718) https://www.ncbi.nlm.nih.gov/nuccore/NC_004718) were downloaded from the NCBI database (https://www.ncbi.nlm.nih.gov/).

For manuscripts utilizing custom algorithms or software that are central to the research but not yet described in published literature, software must be made available to editors and reviewers. We strongly encourage code deposition in a community repository (e.g. GitHub). See the Nature Research guidelines for submitting code & software for further information.

Data

Policy information about availability of data

All manuscripts must include a data availability statement. This statement should provide the following information, where applicable:

- Accession codes, unique identifiers, or web links for publicly available datasets
- A list of figures that have associated raw data
- A description of any restrictions on data availability

Raw data in this study are provided in the Source data. Additional supporting data are available from the corresponding authors upon reasonable request. All requests for raw and analyzed data and materials will be reviewed by the corresponding authors to verify if the request is subject to any intellectual property or confidentiality obligations. Patient-related data not included in the paper were generated as part of clinical examination and may be subject to patient confidentiality. Any data that can be shared will be released via a Material Transfer Agreement.
Reference viral sequences 229E (NC_002645), HKU1 (NC_006577), NL63 (NC_005831), OC43 (NC_006213), MERS (NC_019843), SARS-CoV-1 (NC_004718) and SARS-CoV-2 (MN908947.3) were downloaded from the NCBI database (https://www.ncbi.nlm.nih.gov/).

Field-specific reporting

Please select the one below that is the best fit for your research. If you are not sure, read the appropriate sections before making your selection.

☒ Life sciences ☐ Behavioural & social sciences ☐ Ecological, evolutionary & environmental sciences

For a reference copy of the document with all sections, see nature.com/documents/nr-reporting-summary-flat.pdf

Life sciences study design

All studies must disclose on these points even when the disclosure is negative.

Sample size	Patients were recruited and patient material was banked at the University Hospital Freiburg; inclusion criteria were: (1) 32 health care workers that received a prime and boost vaccination with the mRNA vaccine bnt162b2/Comirnaty, (2) 59 acutely infected and convalescent individuals following a mild course of SARS-CoV-2 infection, SARS-CoV-2 infection was confirmed by positive PCR testing from oropharyngeal swab and/or SARS-CoV-2 spike IgG positive antibody testing, (3) 2 convalescent health care workers following a mild course of SARS-CoV-2 infection that received a single dose of bnt162b2/Comirnaty and (4) 8 age and sex-matched historic controls. No sample size calculations were performed. 32 vaccinatedhealth care workers gave informed consent and were available to donate blood samples. Therefore, similar numbers of COVID-19 convalescents were selected.
Data exclusions	For flow cytometrical analysis, cell populations containing less than 5 cells were excluded. This data exclusion strategy has been applied and validated previously by our group to gain reproducible results in studies investigating virus-specific CD8+ T cells in human viral infections.
Replication	Analyses were performed in independent experiments. Findings were reproducible. Flow cytometry analysis: 5 longitudinally analyzed vaccinees for A*01/S865 (n= 5 at BL, 4 at 3-4 dpp, 5 at 6-8 dpp, 5 at 9-12 dpp, 5 at 13-15 dpp, 2 at 16-18 dpp, 1 at 19-21 dpp, 4 at 3-4 dpb, 3 at 5-6 dpb, 2 at 7-9 dpb, 4 at 13-16 dpb, 4 at 28-30 dpb, 4 at 55-58 dpb and 5 at 80-120 dpb), 5 longitudinally anayzed vaccinees for A*02/S269 (n=5 at BL, 3 at 3-4 dpp, 4 at 6-8 dpp, 4 at 9-12 dpp, 5 at 13-15 dpp, 3 at 16-18 dpp, 2 at 19-21 dpp, 5 at 3-4 dpb, 2 at 5-6 dpb, 1 at 7-9 dpb, 5 at 13-16 dpb, 5 at 28-30 dpb, 4 at 55-58 dpb and 5 at 80-120 dpb) and 4 longitudinally analyzed vaccinees for A*03/S378 (n= 4 at BL, 3 at 3-4 dpp, 4 at 6-8 dpp, 4 at 9-12 dpp, 4 at 13-15 dpp, 1 at 19-21 dpp, 2 at 3-4 dpb, 2 at 5-6 dpb, 3 at 7-9 dpb, 3 at 13-16 dpb, 3 at 28-30 dpb and 4 at 80-120 dpb) in independent experiments (Figure 1, 2, Extended Data Figure 2d, e, 3, 4d, e, f, 5c), 2 longitudinally analyzed convalescent health care workers following a mild course of SARS-CoV-2 infection that received a single dose of bnt162b2/Comirnaty for A*02/S269 (n=2 at convalescent time point prior vaccination, n=2 at 3-4 dpp, n=2 at 8-9 dpp, n=2 at 13-15 dpp and n=2 at 22-23 dpp) in independent experiments (Extended Data Figure 4a, b, c), 8 longitudinally analyzed vaccinees for DRB1*15:01/S236 (n= 8 at BL, 5 at 3-4 dpp, 6 at 6-8 dpp, 7 at 9-12 pp, 6 at 13-15 dpp, 4 at 16-18 dpp, 6 at 3-4 dpb, 4 at 5-6 dpb, 3 at 7-9 dpb, 4 at 13-16 dpb, 7 at 28-30 dpb, 1 at 55-58 dpb and 1 at 84-86 dpb) in independent experiments (Figure 3a, b, c, Extended Data Figure 5e, f, g), 8 cross-sectionally analyzed historic controls (DRB1*15:01/S236) in independent experiments (Extended Data Figure 6d), 8 longitudinally analyzed vaccinees for S1 (n=8 at BL, 8 at 13-15 dpp, 2 at16-18 dpp, 3 at 19-21 dpp, 5 at 2-4 dpp, 3 at 5-6 dpp, 7 at 28-30 dpp) and RBD (n=8 at BL, 8 at 13-15 dpp, 2 at16-18 dpp, 3 at 19-21 dpp, 5 at 2-4 dpp, 3 at 5-6 dpp, 7 at 28-30 dpp), in independent experiments (Figure 3b, f, Extended Data Figure 7c), and 10 donors with a history of natural SARS-CoV-2 infection cross-sectionally for S1 and 10 cross-sectionally for RBD in independent experiments (Figure 3f, g, Extended Data Figure 7c), 5 longitudinally analyzed vaccinees for A*01/S865 (n=5 at 20-40dpb and 4 at 40-80 dpb), 5 longitudinally analyzed vaccinees for A*02/S269 (n=5 at 20-40dpb and 4 at 40-80 dpb), 4 longitudinally analyzed vaccinees for A*03/S378 (n=4 at 20-40dpb and 2 at 40-80 dpb), 9 donors with a history of natural SARS-CoV-2 infection cross-sectionally for A*01/S865 (n=5 at 20-40dpb, 4 at 40-80 dpb), 8 donors with a history of natural SARS-CoV-2 infection cross-sectionally for A*02/S269 (n=4 at 20-40dpb, 4 at 40-80 dpb), 7 donors with a history of natural SARS-CoV-2 infection cross-sectionally for A*03/S378 (n=4 at 20-40dpb, 3 at 40-80 dpb) in independent experiments (Extended Data Figure 8a, b, 9b, c), 11 cross-sectionally analyzed vaccinees for A*01/S865 at 80-120 dpb, 9 cross-sectionally anayzed vaccinees for A*02/S269 at 80-120 dpb, 8 cross-sectionally anayzed vaccinees for A*03/S378 at 80-120 dpb, 10 donors with a history of natural SARS-CoV-2 infection cross-sectionally for A*01/S865 80-120 dpb, 10 donors with a history of natural SARS-CoV-2 infection cross-sectionally for A*02/S269 80-120 dpb and 10 donors with a history of natural SARS-CoV-2 infection cross-sectionally for A*03/S378 80-120 dpb in independent experiments (Figure 4a, b, c, d, Extended Data Figure 9c, 10). ELISA and NT analysis: 8 longitudinally analyzed vaccinees for anti-SARS-CoV-2-S1 IgG (n= 8 at BL, 5 at 3-4 dpp, 7 at 6-8 dpp, 7 at 9-12 dpp, 8 at 13-15 dpp, 4 at 16-18 dpp, 3 at 19-21 dpp, 7 at 3-4 dpb, 4 at 5-6 dpb, 2 at 7-9 dpb, 7 at 13-16 dpb, 7 at 28-30 dpb, 8 at 55-58 dpb and 4 at 84-86 dpb) in independent experiments (Figure 3d), 8 longitudinally analyzed vaccinees for B.1 neutralizing titer (n= 6 at BL, 5 at 3-4 dpp, 6 at 6-8 dpp, 7 at 13-15 dpp, 2 at 19-21 dpp, 6 at 3-4 dpb, 3 at 5-6 dpb, 7 at 13-16 dpb, 7 at 28-30 dpb, 7 at 55-58 dpb and 5 at 84-86 dpb) in independent experiments (Figure 3d, Extended Data Figure 3e), 7 longitudinally analyzed vaccinees for alpha neutralizing titer (n=6 at BL, 6 at 6-8 dpp, 7 at 13-16 dpb, 2 at 19-21 dpp, 6 at 3-4 dpb, 3 at 5-6 dpb, 7 at 13-16 dpb, 5 at 28-30 dpb, 7 at 55-58 dpb and 5 at 84-86 dpb) and 7 longitudinally analyzed vaccinees for beta neutralizing titer

(n=6 at BL, 6 at 6-8 dpp, 7 at 13-16 dpb, 2 at 19-21 dpp, 6 at 3-4 dpb, 3 at 5-6 dpb, 7 at 13-16 dpb, 5 at 28-30 dpb, 7 at 55-58 dpb and 5 at 84-86 dpb) in independent experiments (Extended Data Figure 3d), 16 donors with a history of natural SARS-CoV-2 infection cross-sectionally/longitudinally for B.1 neutralizing titer (n=4 at 20-40 dps, 5 at 40-80 dps and 7 at >80 dps) in independent experiments (Extended Data Figure 7e), 9 longitudinally analyzed vaccinees for anti-SARS-CoV-2 S1 IgM supernatant (n=9 at BL, 9 at 13-15 dpp, 2 at 16-18 dpp, 3 at 19-21 dpp, 5 at 2-4 dpb, 4 at 5-6 dpb and 9 at 28-30 dpb) and 4 donors with a history of natural SARS-CoV-2 infection cross-sectionally for anti-SARS-CoV-2 S1 IgM supernatant (n=9 at BL, 9 at 13-15 dpp, 2 at 16-18 dpp, 3 at 19-21 dpp, 5 at 2-4 dpb, 4 at 5-6 dpb and 9 at 28-30 dpb) and 8 longitudinally analyzed vaccinees for S1 binding IgG production after re-stimulation (n=8 at BL, 8 at 13-15 dpp, 2 at 16-18 dpp, 3 at 19-21 dpp, 5 at 2-4 dpb, 3 at 5-6 dpb and 7 at 28-30 dpb) and 8 donors with a history of natural SARS-CoV-2 infection cross-sectionally in independent experiments (Figure 3g). Overlapping peptides analyses: 16 analyzed vaccinees for 182 overlapping peptides spanning the SARS-CoV-2 Spike sequence (9-59 dpb) in independent experiments (Extended Data Figure 1d).

Randomization

Vaccinated donors and donors with a history of natural SARS-CoV-2 infection were selected based on availability and HLA-typing. To analyze A*01/S865, A*02/S269, A*03/S378 and DRB1*15:01/S236-specific T cells participants needed to be allocated into experimental groups based on their HLA-typing. The covariates age and gender are well-documented: Median age of vaccinated donors was 39,6 years, donors with a history of natural SARS-CoV-2 infection was 47,2 years, donors with a history of natural SARS-CoV-2 vaccination and a single vaccination was 56,5 years, of historic controls 37,6 years. The gender ratio of vaccinated donors was m/f: 19/13, donors with a history of natural SARS-CoV-2 infection was m/f: 31/28, donors with a history of natural SARS-CoV-2 vaccination and a single vaccination was m/f: 1/1, of historic controls m/f: 5/3.

Blinding

Blinding was not applied. Non-objective parameters were not included in the study design. Due to standardized analyses of the flow cytometric data set, biased analysis can be excluded.

Reporting for specific materials, systems and methods

We require information from authors about some types of materials, experimental systems and methods used in many studies. Here, indicate whether each material, system or method listed is relevant to your study. If you are not sure if a list item applies to your research, read the appropriate section before selecting a response.

Materials & experimental systems

n/a	Involved in the study
☐	☒ Antibodies
☒	☐ Eukaryotic cell lines
☒	☐ Palaeontology and archaeology
☒	☐ Animals and other organisms
☐	☒ Human research participants
☒	☐ Clinical data
☒	☐ Dual use research of concern

Methods

n/a	Involved in the study
☒	☐ ChIP-seq
☐	☒ Flow cytometry
☒	☐ MRI-based neuroimaging

Antibodies

Antibodies used

T cell analysis
BD Biosciences:
anti-CCR7-PE-CF594 (150503, 1:50), Cat# 353232
anti-CCR7-BUV395 (3D12, 1:25), Cat# 740267
anti-CD4-BV786 (L200, 1:200), Cat# 563914
anti-CD8-BUV395 (RPA-T8, 1:400), Cat# 563795
anti-CD8-BUV510 (SK1, 1:100), Cat# 563914
anti-CD8-APC (SK-1, 1:200), Cat# 345775
anti-CD11a-BV510 (HI111, 1:25), Cat# 563480
anti-CD28-BV421 (CD28.2, 1:100), Cat# 562613
anti-CD28 pure (CD28.2, 1:1000), Cat# 555726
anti-CD38-APC-R700 (HIT2, 1:400), Cat# 564980
anti-CD38-BUV737 (HB7, 1:200), Cat# 564686
anti-CD39-BV650 (TU66, 33:1), Cat# 563681
anti-CD45RA-BUV496 (HI100, 1:800), Cat# 750258
anti-CD45RA-BUV737 (HI100, 1:200), Cat# 564442
anti-CD69-BUV395 (FN50, 1:50), Cat# 564364
anti-CD107a-APC (H4A3, 1:100), Cat# 560664
anti-CD127-BUV737 (HIL-7R-M21, 1:50), Cat# 612795
anti-CD127-BV421 (HIL-7R-M21, 3:100), Cat# 562436
anti-Granzyme B-PE-CF594 (GB11, 1:100), Cat# 562462
anti-ICOS-BV711 (DX29, 1:100), Cat# 563833
anti-IFN-γ-FITC (25723.11, 1:8), Cat# 340449
anti-IL-21-PE (3A3-N2.1, 1:25), Cat# 560463
anti-PD-1-BV605 (EH12.1, 1:50), Cat# 563245
anti-PD-1-PE-Cy7 (EH12.2H7, 1:200), Cat# 561272
anti-PD-1-BV786 (EH12.1, 1013122, 3:100), Cat# 563789
anti-T-BET-PE-CF594 (O4-46,93533305, 3:100), Cat# 562467
anti-TNF-PE-Cy7 (Mab11, 1:400), Cat# 557647
ViaProbe (7-AAD, 1:33), Cat# 555816

BioLegend
anti-BCL-2-BV421 (100, 1:200), Cat# 658709
anti-CCR7-BV785 (G043H7, 1:50), Cat# 353230
anti-CD4-AlexaFluor700 (RPA-T4, 300526, 1:200), Cat# 300526
anti-CD25-BV650 (BC96, 1:33), Cat# 302633
anti-CD57-BV605 (QA17A04, 1:100), Cat# 563895
anti-CD127-BV605 (A019D5, 3:100), Cat# 351334
anti-CXCR3-PerCP-Cy5.5 (G025H7, 1:33), Cat# 353714
anti-CXCR3-BV510 (G025H7, 3:100), Cat# 353726
anti-CXCR5-BV421 (J252D4, 1:100), Cat# 356920
anti-IL-2-PerCP-Cy5.5 (MQ1-17H12, 1:100), Cat# 500322
anti-Ki67-BV711 (Ki-67, 1:200), Cat# 350516
anti-Ki67-PE-Cy7 (Ki67, 1:200), Cat# 350504

Cell Signaling,
anti-TCF1-AlexaFluor488 (C63D9, 1:100), Cat# 6444

eBioscience
anti-CD14-APC-eFluor780 (61D3, 1:400), Cat# 47-0149-42
anti-CD19-APC-eFluor780 (HIB19, 1:400), Cat# 47-0199
anti-CD27-FITC (0323, 1:100), Cat# 11-0279
anti-KLRG1-BV711 (13F12F2, 1:50), Cat# 67-9488-42
anti-T-BET-PE-Cy7 (4B10, 1:200), Cat# 25-5825
anti-TOX-eFluor660 (TRX10, 1:100), Cat# 50-6502
anti-EOMES-PerCP-eF710 (WD1928, 1:50), Cat# 46-4877-42
Viability Dye (APC-eFluor780 1:200, 1:400) Cat# 65-0865

Invitrogen

anti-CD45RA-PerCP-Cy5.5 (HI100, 3:100), Cat# 45-0458-42

B cell analysis

BioLegend
anti-CD20-BV510 (2H7, 1:80), Cat# 302340
anti-IgM-BV605 (MHM-88, 1:200), Cat# 314524
anti-CD24-FITC (ML5, 1:1000), Cat# 334103
anti-CD71-FITC (CY1G4, 1:1000), Cat# 334103
anti-CD95-PE-Dazzle594 (DX2, 1:50), Cat# 305634
anti-CD38-PE-Cy7 (HB-7), Cat# 356608
anti-BAFF-R-AF647 (11C1, 1:100), Cat# 316914
anti-CD19-APC-Cy7 (HIB19, 1:150), Cat# 302218
Zombie NIR Fixable Viability Kit (1:800), Cat# 423106

BD Biosciences
anti-IgG-BV650 (G18-145, 1:600), Cat# 740596
anti-CD27-BV786 (L128, 1:100), Cat# 563327
anti-CD69-BV480 (FN50, 1:200), Cat# 747519

Jackson ImmunoResearch
anti-IgA-PerCP (polyclonal, 1:200), Cat# 109-125-011

Invitrogen
anti-CD3-SB436 (OKT3, 1:200), Cat# 62-0037-42
anti-CD33-SB436 (WM-53, 1:50) Cat# 62-0338-42
anti-IgD-PerCP-eFluor 710 (IA6-2, 1:200), Cat# 46-9868-42

Validation

All antibodies were obtained from commercial cendors and we based specificity on descriptions and information provided in corresponding data sheets available and provided by the manufacturers. Standardized analysis in different cohorts, antibody titration on PBMCs including unstained controls, comparisons of different antibody clones and conjugates and validated by publications:
CCR7, clone 3D12 and 150503: antibody titration on PBMCs; control clone G043H7; validated with respect to differential expression of naïve and non-naïve T cell subpopulations
CD4, clone L200 and RPA-T4: antibody titration on PBMCs; control clones SK3; using B cells as negative control
CD8, clone SK1 and RPA-T8: antibody titration on PBMCs; control clone GHI/75; using B cells as negative control
CD11, clone HI111: antibody titration on PBMCs, control clones TS2/4 and G43-25B; validated with respect to differential expression of activated and non-activated T cell subpopulations
CD27, clone O323: antibody titration on PBMCs; control clone L128; validated with respect to differential expression of naïve and non-naïve T cell subpopulations
CD28,clone CD28.2: antibody titration on PBMCs; control clone B-T3; validated with respect to differential expression of naïve and non-naïve T cell subpopulations
CD45RA, clone HI100: antibody titration on PBMCs; validated with respect to differential expression of naïve and non-naïve T cell subpopulations
CD69, clone FN50: antibody titration on PBMCs; validated with respect to differential expression of activated and non-activated T cell subpopulations
CD107a, clone H4A3: antibody titration on PBMCs; validated with respect to differential expression of activated and non-activated T

cell subpopulations
CD127, clone HIL-7R-M21 and A019D5: antibody titration on PBMCs; control clone eBioRDR5; validated with respect to differential expression of naïve and non-naïve T cell subpopulations
Granzyme B, clone GB11: antibody titration on PBMCs; polyclonal antibody as control; validated with respect to differential expression of activated and non-activated T cell subpopulations
IFNɣ, clone 25723.11: antibody titration on PBMCs; control clone 4S.B3; validated with respect to differential expression of activated and non-activated T cell subpopulations
IL-21, clone 3A3-N2.1: antibody titration on PBMCs; validated with respect to differential expression of activated and non-activated T cell subpopulations
PD-1, clone EH12.2H7 and EH12.1: antibody titration on PBMCs; control clone eBioJ105; validated with respect to differential expression of naïve and non-naïve T cell subpopulations
TNF, clone MAb11: antibody titration on PBMCs; validated with respect to differential expression of activated and non-activated T cell subpopulations
Bcl-2 , clone 100: antibody titration on PBMCs; validated with respect to differential expression of naïve and non-naïve T cell subpopulations
CD25, clone BC96: antibody titration on PBMCs; control clone M-A251; validated with respect to differential expression of activated and non-activated T cell subpopulations
CD38, clone HB7 and HIT2: antibody titration on PBMCs; control clone HIT2.1; validated with respect to differential expression of naïve and non-naïve T cell subpopulations
CD39, clone TU66: antibody titration on PBMCs; control clones eBioA1 and A1; validated with respect to differential expression of naïve and non-naïve T cell subpopulations
CD57, clone QA17A04: antibody titration on PBMCs; control clone NK-1; validated with respect to differential expression of naïve and non-naïve T cell subpopulations
CXCR3, clone G025H7: antibody titration on PBMCs; control clone 1C6/CXCR3; validated with respect to differential expression of activated and non-activated T cell subpopulations
IL-2, clone MQ1-17H12: antibody titration on PBMCs; validated with respect to differential expression of activated and non-activated T cell subpopulations
TCF-1, clone C63D9: antibody titration on PBMCs; control clone 7F11A10; validated with respect to differential expression of naïve and non-naïve T cell subpopulations
Eomes, clone WD1928: antibody titration on PBMCs; validated with respect to differential expression of naïve and non-naïve T cell subpopulations
CD14 , clone 61D3: antibody titration on PBMCs; control clones M5E2 and MϕP9; using T cell populations as negative control
CD19, clone HIB19: antibody titration on PBMCs; control clone SJ25C1; using T cell populations as negative control
T-bet, clone O4-46: antibody titration on PBMCs; control clones 4B10; validated with respect to differential expression of naïve and non-naïve T cell subpopulations
KLRG1, clone 13F12F2: antibody titration on PBMCs; validated with respect to differential expression of naïve and non-naïve T cell subpopulations
TOX1, clone TRX10: antibody titration on PBMCs; control clone REA473; validated with respect to differential expression of naïve and non-naïve T cell subpopulations
CXCR5, clone J252D4: antibody titration on PBMCs; control clone MU5UBEE; validated with respect to differential expression of naïve and non-naïve T cell subpopulations
ICOS, clone DX29: antibody titration on PBMCs; control clone ISA-3; validated with respect to differential expression of naïve and non-naïve T cell subpopulations
Ki67, clone Ki67: antibody titration on PBMCs; control clone B56; validated with respect to differential expression of naïve and non-naïve T cell subpopulations
Viability Dye was titrated on PBMCs; validated with respect to differential staining of live and dead cell populations
CD20, clone 2H7: Titration on fresh and cryopreserved PBMCs, staining compared to clone JDC-10 and polyclonal anti-IgM Ab (Jackson ImmunoResearch)
IgM clone MHM-88: Titration on fresh and cryopreserved PBMCs, staining compared to clone JDC-10 and polyclonal anti-IgM Ab
CD71 clone CY1G4: Titration on fresh PBMCs and in vitro activated B cells
CD95 clone DX2: Titration on fresh PBMCs and in vitro activated B cells
CD38 clone HB-7: Titration on fresh and cryopreserved PBMCs; staining compared to clone HIT2
BAFF-R clone 11C1: Titration on cryopreserved PBMCs
CD19 clone HIB19: Titration on cryopreserved PBMCs
IgG clone G18-145: Titration on fresh and cryopreserved PBMCs, staining compared to clone JDC-10 and polyclonal anti-IgG Ab
CD27 clone L128: Titration on fresh and crypreserved PBMCs, staining compared to clone LG.3A10
IgA (polyclonal): Titration on fresh and cryopreserved PBMCs
IgD clone IA6-2: Titration on fresh and cryopreserved PBMCs
CD24, clone ML5: Titration on fresh PBMCs and in vitro activated B cells
CD3, clone OKT3:Titration on cryopreserved PBMCs
CD33, clone WM-53: Titration on cryopreserved PBMCs
CD69, clone FN50: Titration on in vitro activated B cells

Human research participants

Policy information about studies involving human research participants

Population characteristics

32 health care workers that received a prime and boost vaccination with the mRNA vaccine bnt162b2/Comirnaty, 59 acutely infected and convalescent individuals following a mild course of SARS-CoV-2 infection, 2 convalescent health care workers following a mild course of SARS-CoV-2 infection that received a single dose of bnt162b2/Comirnaty and 8 historic controls, carrying either of the following HLA alleles: A*01:01, -A*02:01, -A*03:01, DRB1*15:01 were recruited at the University Hospital Freiburg. Median age of vaccinated donors was 39,6 years, donors with a history of natural SARS-CoV-2 infection was 47,2 years, donors with a history of natural SARS-CoV-2 vaccination and a single vaccination was 56.5 years, of historic

	controls 37,6 years. The gender ratio of vaccinated donors was m/f: 19/13, donors with a history of natural SARS-CoV-2 infection was m/f: 31/28, donors with a history of natural SARS-CoV-2 vaccination and a single vaccination was m/f: 1/1, of historic controls m/f: 5/3.
Recruitment	Vaccinated donors as well as SARS-CoV-2-infected and SARS-CoV-2-convalescent patients were recruited at the University Hospital Freiburg (in- and outpatient section); self-selection bias or other biases can be excluded since several people were included in the recruitment. Samples were banked and retrospectively selected according to the following inclusion criteria: HLA-A*01:01, -A*02:01, -A*03:01, DRB1*15:01. Banked samples from sex-, age- and HLA-matched historic controls were retrospectively selected.
Ethics oversight	Written informed consent was obtained from all participants and the study was conducted according to federal guidelines, local ethics committee regulations (Albert-Ludwigs-Universität, Freiburg, Germany; vote #: 322/20, #21-1135 and 315/20) and the Declaration of Helsinki (1975).

Note that full information on the approval of the study protocol must also be provided in the manuscript.

Flow Cytometry

Plots

Confirm that:

☒ The axis labels state the marker and fluorochrome used (e.g. CD4-FITC).

☒ The axis scales are clearly visible. Include numbers along axes only for bottom left plot of group (a 'group' is an analysis of identical markers).

☒ All plots are contour plots with outliers or pseudocolor plots.

☒ A numerical value for number of cells or percentage (with statistics) is provided.

Methodology

Sample preparation	Cryopreserved isolated human PBMCs were thawed and prepared for flow cytometry or in vitro expansion described in the methods section
Instrument	FACSCanto II, LSRFortessa (BD, Germany), Cytek Aurora (Cytek) or CytoFLEX (Beckman Coulter), Tecan (LifeScience)
Software	FlowJo_v10.6.2 (Treestar), R version 4.0.2 using the Bioconductor (version: Release (3.11)) .
Cell population abundance	Abundance of SARS-CoV-2-specific T cells are low (<10^-4 %)
Gating strategy	CD8+ T cells: Lymphocytes gated on FSC-A and SSC-A, Doublet exclusion on FSC-A and FSC-H and FSC-A and FSC-W, Exclusion of dead cells, B cells and monocytes, Gating on CD8+ cells, Exclusion of naive cells (CCR7+CD45RA+), Gating of SARS-CoV-2-specific CD8+ T cells via tetramers described in methods part.

☒ Tick this box to confirm that a figure exemplifying the gating strategy is provided in the Supplementary Information.
